# Cell cycle dynamics control fluidity of the developing mouse neuroepithelium

**DOI:** 10.1038/s41567-023-01977-w

**Published:** 2023-04-06

**Authors:** Laura Bocanegra-Moreno, Amrita Singh, Edouard Hannezo, Marcin Zagorski, Anna Kicheva

**Affiliations:** 1 Institute of Science and Technology Austria, Klosterneuburg, Austria; 2 Institute of Theoretical Physics and Mark Kac Center for Complex Systems Research, Jagiellonian University, Krakow, Poland

**Keywords:** Biophysics, Biological physics

## Abstract

As developing tissues grow in size and undergo morphogenetic changes, their material properties may be altered. Such changes result from tension dynamics at cell contacts or cellular jamming. Yet, in many cases, the cellular mechanisms controlling the physical state of growing tissues are unclear. We found that at early developmental stages, the epithelium in the developing mouse spinal cord maintains both high junctional tension and high fluidity. This is achieved via a mechanism in which interkinetic nuclear movements generate cell area dynamics that drive extensive cell rearrangements. Over time, the cell proliferation rate declines, effectively solidifying the tissue. Thus, unlike well-studied jamming transitions, the solidification uncovered here resembles a glass transition that depends on the dynamical stresses generated by proliferation and differentiation. Our finding that the fluidity of developing epithelia is linked to interkinetic nuclear movements and the dynamics of growth is likely to be relevant to multiple developing tissues.

## Abstract (web summary)

Developing tissues undergo rheology transitions that are often linked to cell–cell adhesion. Now, tissue fluidity is linked to interkinetic nuclear movements and tissue growth.

## Main

Cells within developing tissues reorganize at the same time as tissue growth takes place. The extent and dynamics of cell rearrangements can substantially change during tissue development^1,2^, reflecting solid–fluid transitions in the physical properties of tissues. In most cases, these transitions have been proposed to result from alterations in cell density, cell motility, internal myosin- and/or cadherin-mediated adhesion forces at cell junctions, or external mechanical forces^2–8^. Cell rearrangements have also been shown in theory and in some experimental situations to depend on active stresses within tissues, such as the ones generated by cell division^9–12^. Yet, in many cases, the dynamics of cell rearrangements and the factors that control them are poorly understood.

The spinal cord of amniotes develops from a flat epithelial sheet—the neural plate—that folds to form a closed neural tube^13^. These morphogenetic changes are accompanied by cell intercalations and convergent extension, which are mediated by planar cell polarity and actomyosin-dependent contractility of the apical adherens junctions, as well as basolateral protrusive activity^14,15^. However, whether these are the only factors contributing to cell rearrangements in the neuroepithelium is an open question. Furthermore, the quantitative dynamics of cell rearrangements during development remain unclear. Here we use highly resolved clonal analysis to measure the rate of cell rearrangements in the mouse neuroepithelium over time, thus inferring the long-term rheological properties of the tissue. We further propose a theoretical framework for how active stresses generated during tissue growth contribute to cell rearrangements.

## Cell rearrangements decline over time

**Fig. 1:**
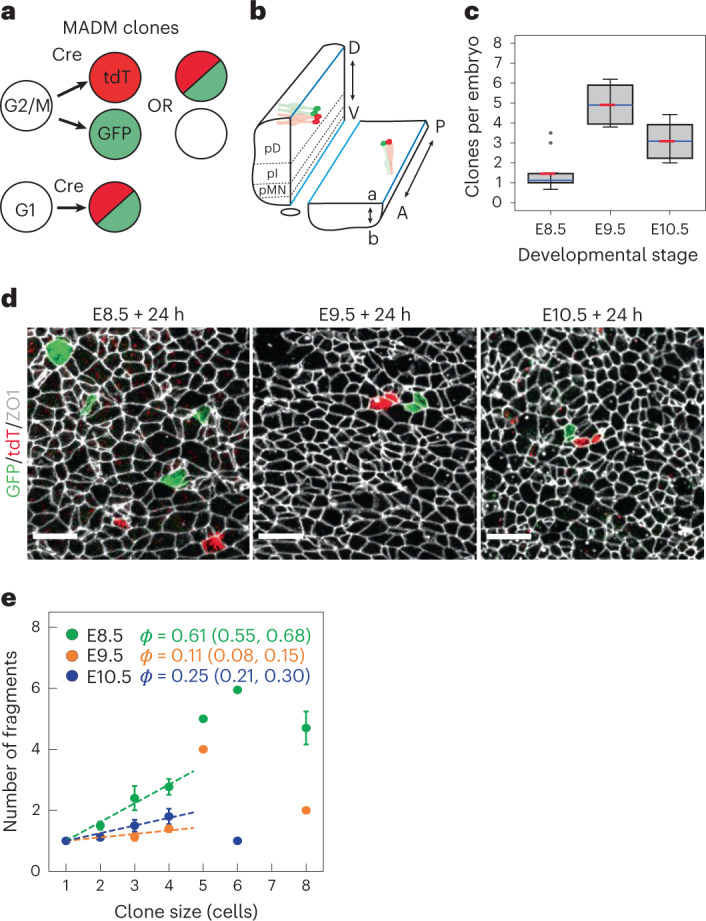
Clonal analysis reveals the dynamics of cell rearrangements in the developing spinal cord. **a**, Clones generated with MADM labelling can comprise cells labelled with EGFP, tdTomato (tdT) or both depending on the type of recombination (Methods). **b**, To analyse the cellular and clonal shapes at the apical surface, the neural tube was opened by dissection along the dorsal and ventral midlines (blue lines). D–V patterning results in the formation of distinct progenitor domains along the D–V axis: pD, pI and pMN are denoted. A, anterior; P, posterior; a, apical; b, basal; D, dorsal; V, ventral. **c**, Mean number of MADM clones per embryo across litters (*n* = 10, 4 and 6 litters at E8.5, E9.5 and E10.5, respectively). 25–75 percentile (box), median (blue), mean (red), highest/lowest observations without outliers (whiskers). Two sided *t-*tests: *P* = 0.005 (E8.5 versus E9.5); *P* = 0.051 (E9.5 versus E10.5); *P* = 0.011 (E8.5 versus E10.5). **d**, MADM clones induced at the indicated stages and analysed 24 h later. Scale bars, 10 μm. **e**, Mean number of fragments per clone for a given clone size ± s.e.m. Clones analysed 24 h after injection at the indicated stages. Both EGFP and tdTomato cells were included in the analysis. Corresponding fragmentation coefficient *ϕ* with 95% CI was obtained using linear fit to the data for clones ≤4 cells (dashed lines). Sample sizes (for **c** and **e**). E8.5, *n* = 46 clones; E9.5, *n* = 87 clones; E10.5, *n* = 94 clones (Supplementary Table 1). Source data

To quantitatively measure cell rearrangements in the neural tube without the risk of perturbing the native mechanical environment of embryo growth in utero, we used clonal labelling to track how the positions of daughter cells that are initially adjacent change with respect to each other over time (Fig. 1a). A key aspect to achieve reliable tracing is the sparseness of labelling. Mosaic analysis with double markers (MADM)^16,17^ is a two-colour labelling system known for its sparseness. Therefore, we used Sox2–CreERT2-induced MADM recombination (Fig. 1a and Supplementary Fig. 1a,b) to label cells with very low probability—we detected between one and five clones per spinal cord (Fig. 1c).

We induced MADM clonal labelling by injecting pregnant mothers with tamoxifen at embryonic days E8.5, E9.5 and E10.5, and harvested the embryos 24 h later. The cytosolic fluorescent reporters allow the labelled cells to be detected at the apical surface of the epithelium (Fig. 1b,d). In addition, immunostaining for the tight junction marker ZO1 allows us to segment individual cells and precisely determine the number and neighbour relationships of the labelled cells.

We focused our analysis on clones in the dorsal (pD) and intermediate (pI) progenitor domains, which span more than half the D–V length of the neural tube (Fig. 1b). The mean clone sizes of MADM clones decline from 4.1 ± 0.3 cells per clone at E8.5 to 2.1 ± 0.1 cells per clone at E10.5 (Supplementary Fig. 1c). This reflects a twofold decline in the tissue growth rate (from 0.087 ± 0.009 h^−1^ to 0.046 ± 0.004 h^−1^), which is consistent with previous estimates^18^ (Supplementary Fig. 1d and Methods). The clone size distribution at E8.5 further shows that 2, 4 and 8 cell clones are the most abundant, indicating that cells divide up to three times and without substantial progenitor loss (Supplementary Fig. 1e). At E9.5 and E10.5, larger clones are progressively under-represented, consistent with a longer cell cycle length and loss of progenitors due to terminal differentiation at these stages. Together, these observations indicate that the MADM clones accurately reflect the dynamics of tissue growth.

We next analysed the clonal shapes to estimate the extent of cell rearrangements. In many tissues, such as the *Drosophila* wing disc or mouse skin, uniform tissue growth with minimal cell rearrangements results in the formation of coherent clones^19,20^. By contrast, cell rearrangements cause clone fragmentation, where subsets of labelled cells are surrounded by non-labelled neighbours. We, therefore, used the number of fragments per clone as a readout of cell rearrangements. To exclude the effects of clone size, we measured the fragments for clones of a given size. The number of fragments linearly depends on the clone size for small clone sizes (≤4 cells) for which reliable statistics can be obtained (Fig. 1e). This allows us to define the fragmentation coefficient *ϕ* as the slope of a linear fit to the number of fragments as a function of clone size (for clone sizes ≤4 cells). We found that MADM clones labelled at E9.5 and E10.5 had very few fragments, corresponding to *ϕ* = 0.11 (95% confidence interval (CI) of 0.08 and 0.15) and 0.25 (95% CI of 0.21 and 0.30), respectively. By contrast, clones labelled at E8.5 were highly fragmented with *ϕ* = 0.61 (95% CI of 0.55 and 0.68) (Fig. 1e).

Consistent with their higher fragmentation, clones labelled at E8.5 had dispersed at a larger maximum distance from the clone centroid, namely, 10.2 ± 1.4 μm, whereas clones labelled at E9.5 and E10.5 dispersed up to 3.3 ± 0.4 and 3.0 ± 0.8 μm, respectively (Supplementary Fig. 1f). The dispersal of cells was nearly isotropic with respect to the clone centre, with the exception of clones in the motor neuron progenitor (pMN) domain, which have a larger A–P/D–V aspect ratio compared with clones in other domains at E10.5 of development (Supplementary Fig. 1g). This effect is consistent with previous observations and is related to the differentiation dynamics in the pMN domain^21^. Altogether, these results indicate that cell rearrangements occur frequently before E9.5 and significantly decline at later stages.

## Tissue fluidity at high junctional tension and contractility

**Fig. 2:**
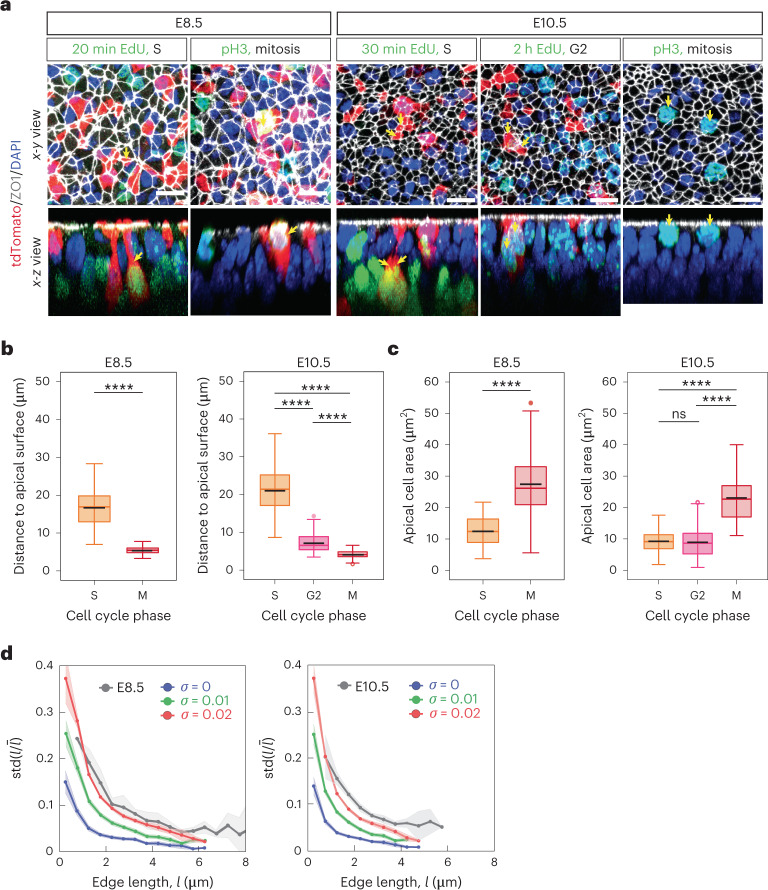
IKNM and cell edge fluctuations in the neuroepithelium at E8.5 and E10.5 of development. **a**, Apical (*x*–*y*) and orthogonal (*x*–*z*) views of neuroepithelial cells in S, G2 and M phases at E8.5 and E10.5. EdU pulses with defined length and pH3 staining were used to distinguish nuclei in the indicated cell cycle phases. Sparse tdTomato labelling was used to track the cell bodies and associate nuclei with the respective apical surfaces (yellow arrows). Scale bars, 10 µm. **b**, Distance from nuclei centres to the apical surface (ZO1). Mitotic nuclei are close to the apical surface (ZO1), whereas S and G2 nuclei are located more basally. **c**, Apical area of cells in the indicated cell cycle phases. In **b** and **c**, 25–75 percentile (box), median (coloured line), mean (black line), highest/lowest observations without outliers (whiskers). Pairwise comparisons two-sided *t-*test: ****P < 0.0001; ns, not significant, *P* > 0.05. Sample sizes (number of cells): E8.5, S (*n* = 172); M (*n* = 179); E10.5, S (*n* = 197); G2 (*n* = 147), M (*n* = 144) (Supplementary Table 1). **d**, Standard deviation of the relative edge length ($l/\bar l$) over a 20 min time interval as a function of absolute edge length *l* (binned in 0.5 μm bins) for simulations with different levels of noise (*σ* = 0, 0.01 and 0.02 with *n* = 1,900, 1,827 and 1,687 edges, respectively; Methods) and in time-lapse images of ZO1–GFP-expressing neuroepithelia (E8.5, *n* = 309; E10.5, *n* = 387; Supplementary Table 1). Shaded regions, 95% CI. Source data

To investigate how the high extent of cell rearrangements at early developmental stages is achieved, we used a two-dimensional vertex model of the apical surface of the neuroepithelium^21,22^. In this model, polygonal cells change neighbours by a process called T1 transition, in which an edge initially shared between two adjacent cells collapses and subsequently reforms in a different orientation, leading to the separation of the cells. The packing geometry of cells in vertex models depends on the normalized tension (${{{\bar{\varLambda }}}}$) and normalized contractility (${{{\bar{\varGamma }}}}$) parameters. In the classical vertex model formulation^22^, cells have a constant target area. By contrast, in our model, the target area depends on the cell cycle time^21^. This reflects the fact that cells in pseudostratified neural epithelia undergo interkinetic nuclear movements (IKNMs) during the cell cycle. In these movements, the position of the nucleus along the apicobasal axis of cells may affect the apical cell surface area. To verify that the IKNM effect we implemented in the model reflects the actual apical-area cell cycle dynamics in the tissue, we measured the distribution of cell areas as a function of cell cycle phase at E8.5 and E10.5. To do this, we used short (20–30 min) EdU pulse labelling to mark the S-phase nuclei, 2 h EdU pulse to mark the G2 nuclei and phospho–histone 3 staining to mark cells undergoing mitosis (Fig. 2a and Methods). We combined EdU/pH3 immunostaining with sparse mosaic cytosolic tdTomato labelling to identify individual cell bodies, and with ZO1 immunostaining to measure the apical surface areas that correspond to specific nuclei. This analysis confirmed that the position of the nucleus relative to the apical surface changes with the cell cycle at both E8.5 and E10.5, reflecting the fact that the nuclei undergo IKNM (Fig. 2b). Furthermore, consistent with the model, we found that cells in mitosis have more than twofold higher mean apical cell area than cells in S phase, whereas the mean apical areas of cells in S and G2 phases were similar (Fig. 2c).

In other epithelia, fluctuations in the levels of myosin activity at cell junctions cause variation in the line tension and edge lengths of cells on a timescale of seconds to minutes^23^. We reasoned that a similar effect might occur in the neuroepithelium. We, therefore, introduced line-tension fluctuations in the model as an Ornstein–Uhlenbeck process. To this end, we introduced a noise term in ${{{\bar{\varLambda }}}}$, drawn from a Gaussian distribution with characteristic deviation time *σ* and temporal correlation time *τ* (Methods). Increasing values of *σ* shifted the distribution of edge length fluctuations in the simulations (Fig. 2d). Hence, to obtain an experimental estimate of *σ*, we performed short-term live imaging of ZO1–GFP-expressing neuroepithelia at E8.5 and E10.5 of development (Methods and Supplementary Video 1). Although this procedure requires neural-tube dissection and the tissues can only be maintained live for 1–2 h, this approach provides an estimate of the variations in edge lengths that occur on shorter timescales. We observed that the distribution of edge length deviation during a 20 min interval corresponds most closely to simulations with *σ* = 0.02 at both E8.5 and E10.5 (Fig. 2d); hence, we used this value of *σ* in our subsequent analysis.

**Fig. 3:**
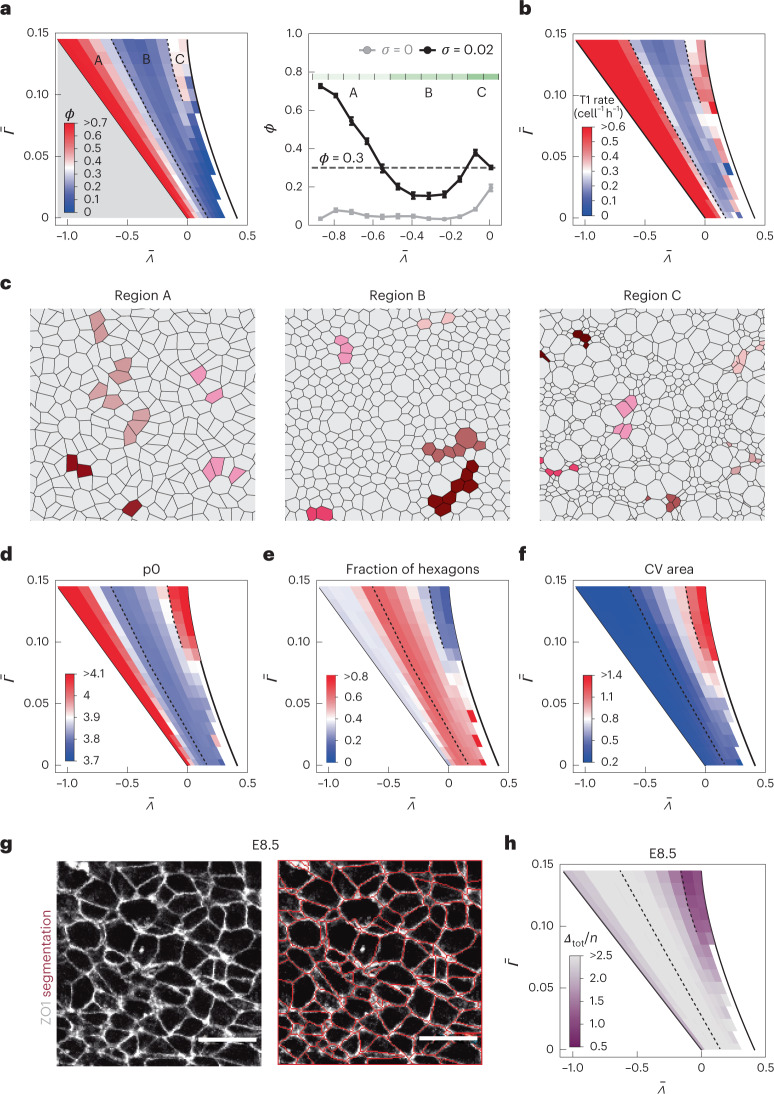
Novel regime of extensive cell rearrangements at high tension and contractility in the early-stage neuroepithelium. **a**, Left: fragmentation coefficient *ϕ* for different values of ${{{\bar{\varLambda }}}}$ and ${{{\bar{\varGamma }}}}$, *k*_p_ = 0.09 h^−1^, *k*_n_ = 0 h^−1^, *σ* = 0.02. The dashed lines correspond to *ϕ* = 0.3 and delineate regions A, B and C. The grey region corresponds to the fluid ground state of the model, and the white region denotes an unstable region due to area collapse. Right: also, *ϕ* for ${{{\bar{\varGamma }}}}$ = 0.12. Error bars, standard error; *n* = 10 simulations. The green-shaded regions denote regions A, B and C. Also, *ϕ* for simulation with no noise (*σ* = 0) is shown for comparison. **b**, Mean rate of T1 transition events (cell^−1^ h^−1^) across the (${{{\bar{\varLambda }}}}$,${{{\bar{\varGamma }}}}$) parameter space. **c**, Snapshots cropped from simulations of regions A, B and C ($\bar\varGamma$ = 0.12 and ${{{\bar{\varLambda }}}} =$ −0.711, −0.393 and −0.074, respectively). *k*_p_ = 0.09 h^−1^, *k*_n_ = 0 h^−1^. Example clones are displayed in different colours. Note that the shape of the simulated tissues changes over time (Supplementary Videos 2–4). **d**–**f**, Mean cell shape index (**d**), fraction of hexagons (**e**) and coefficient of variation of apical cell areas (**f**) for ten simulations per ($\bar{\varLambda}$, $\bar{\varGamma}$) parameter set. **g**, Apical view of the epithelium with ZO1 immunostaining. Cell segmentation (red traces). Scale bars, 10 μm. **h**, Difference between the cumulative distribution of cell shape descriptors *p*_0_, *ϵ*, *α*, *hex*, *p*_0CV_, *ϵ*_CV_, *A*_CV_ and *P*_CV_ (Supplementary Table 3) in the simulations and experimental data. Source data

**Extended Data Fig. 1:**
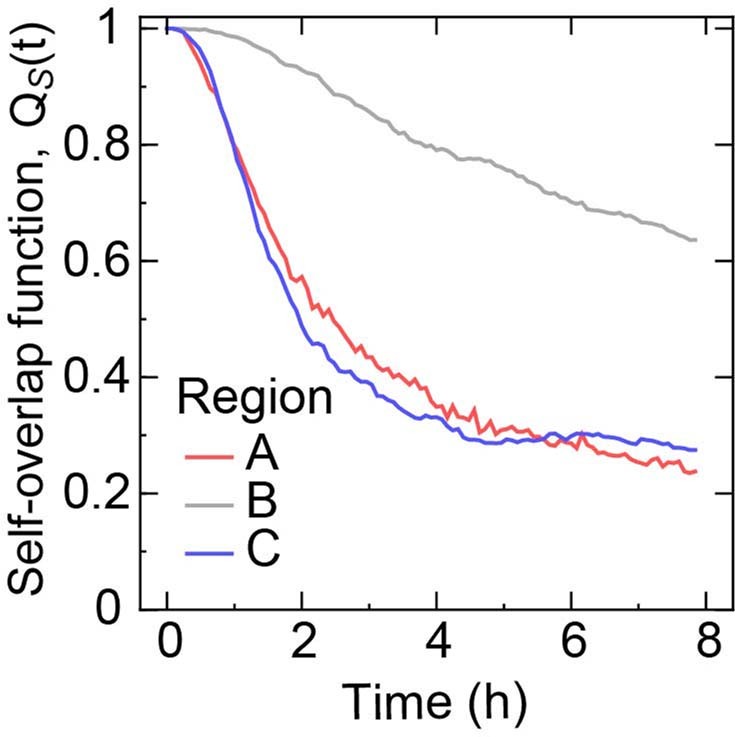
Self-overlap function for regions A, B, C. Q_s_(t) quantifies the fraction of cells that remain within a characteristic distance (approximately one cell radius) after time *t*, after the displacement due to growth has been accounted for (Methods). Thus, fewer cells have changed their relative position in the tissue in region B compared to regions A and C. Single simulation for each region at ${{{\bar{\mathrm \Gamma }}}}$ = 0.12 and varied line tension ${{{\bar{\mathrm \Lambda }}}}$ = −0.711 (Region A, n = 702 cells), ${{{\bar{\mathrm \Lambda }}}}$ = −0.393 (Region B, n = 2546 cells), and ${{{\bar{\mathrm \Lambda }}}}$ = −0.074 (Region C, n = 1805 cells). Only cells that have not divided within 8 h are considered. Source data

To determine the model parameters that reproduce the experimentally observed clone fragmentation, we performed a systematic screening of the intermediate region of the ${{{\bar{\varLambda }}}}$–${{{\bar{\varGamma }}}}$ parameter space, where the network configuration is expected to be the most similar to epithelial tissues^22,24^ (Supplementary Table 2). We used a proliferation rate of 0.09 h^−1^ (equivalent to a cell cycle length of ~8 h), which corresponds to the experimentally measured value at E8.5 (ref. ^18^ and Supplementary Fig. 1d). We traced clones in silico for 16 h, which corresponds to the duration of Cre activity in experiments (Supplementary Fig. 1b and Methods). The model revealed that the fragmentation coefficient varies across the (${{{\bar{\varLambda }}}}$,${{{\bar{\varGamma }}}}$) parameter space (Fig. 3a). In particular, *ϕ* changes non-monotonically along the ${{{\bar{\varLambda }}}}$ axis: it decreases, reaches a local minimum and subsequently increases with increasing values of ${{{\bar{\varLambda }}}}$ (Fig. 3a). To qualitatively capture these differences, we defined an arbitrary threshold value of *ϕ* = 0.3, which subdivides the parameter space into three subregions. We refer to these as regions A, B and C (Fig. 3a). Regions A and C have high fragmentation (*ϕ* ≥ 0.3) and high T1 transition rate, whereas region B has low fragmentation (*ϕ* < 0.3) and low T1 transition rate (Fig. 3a,b and Supplementary Videos 2–4). To further characterize the differences between regions, we compared the profiles of the self-overlap function^25,26^, which quantifies the fraction of cells that remain within approximately a cell radius of their relative initial position in the tissue. We found that these profiles are distinct in regions A and C compared with region B (Extended Data Fig. 1). Differences in the shape of the self-overlap function have been associated with glassy dynamics in vertex models^26^, suggesting that the differences between regions A, C and B represent transitions between fluid-like and solid-like states. Previous studies of vertex models have revealed that a density-independent fluid-to-solid phase transition^1,7,22^ characterized by a change in cell shape index occurs in a similar position in the parameter space to the transition between regions A and B that we observe. By contrast, the high rate of T1 transitions in region C has not been previously observed and is surprising, given that the ground state of the model in this region is solid^22,24^.

The high fragmentation coefficient that we observed at E8.5 (Fig. 1e) is consistent with both high fragmentation regions A and C. Hence, more than one mechanism, captured by either region A or C, could explain how the high fragmentation rates are achieved at early developmental stages. To distinguish potential mechanisms and understand how fragmentation is achieved in the E8.5 neural tube, we compared the cell shapes in simulations of regions A versus C (Fig. 3c–f and Supplementary Fig. 2). Several first-order descriptors of cell shapes (Supplementary Table 3) were similar between regions A and C. For instance, these regions were characterized by high cell shape index and low packing order, measured by the fraction of hexagons, which are indicators of tissue fluidity^7^ (Fig. 3d,e). By contrast, a subset of cell shape descriptors differed between regions A and C. These included the coefficients of variation (CV) of the cell area, perimeter and elongation, as well as the area-ratio slope (Supplementary Fig. 2). The most striking difference between regions A and C was that only region C had high cell area CV, whereas in region A, the cell areas were nearly uniform (Fig. 3f).

**Extended Data Fig. 2:**
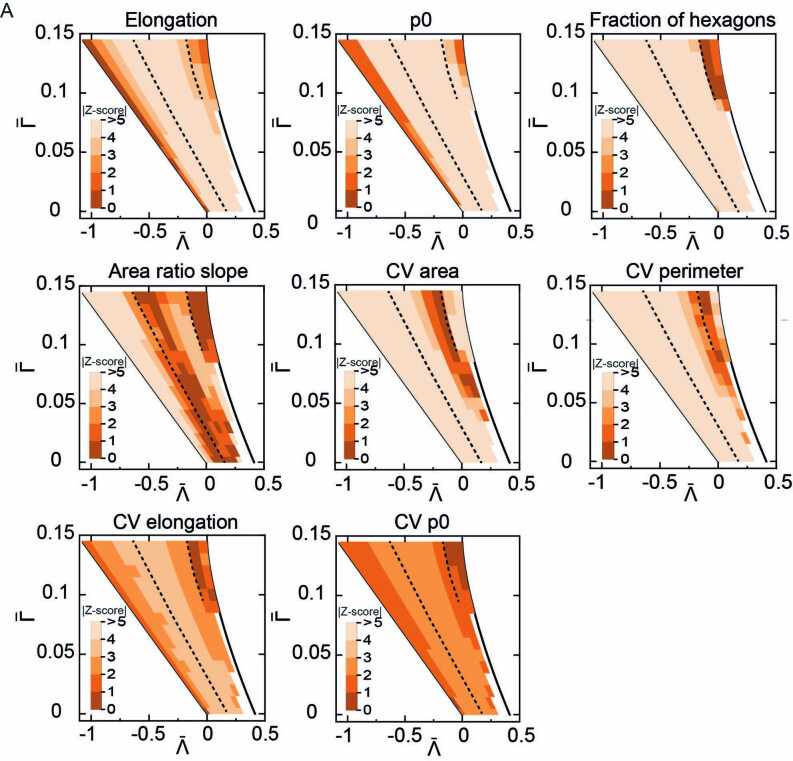
Comparison of cell shape descriptors between experimental data at E8.5 and simulations. Difference (absolute Z-score values) between experimental data at E8.5 and simulations in units of standard deviations of the experimental data separately for each cell shape descriptor (defined in Table S3) across the (${{{\bar{\mathrm \Lambda }}}}$,${{{\bar{\mathrm \Gamma }}}}$) parameter space (see Methods). Low values indicate good agreement between simulation and experimental estimates. The dashed lines correspond to *ϕ* = 0.3 and delineate regions A, B and C (see Fig. 3A). Source data

Comparisons of cell shapes between model and experimental data have been used to infer the mechanical parameters of tissues^27,28^. Therefore, to determine the (${{{\bar{\varLambda }}}}$, ${{{\bar{\varGamma }}}}$) parameter region characteristic of the E8.5 neuroepithelium, we immunolabelled the tight junctions in E8.5 neural plates and segmented the cell shapes (Fig. 3g). We found that for most cell shape descriptors, the best correspondence between data and simulations is in region C (Extended Data Fig. 2). A simultaneous comparison of a set of several descriptors confirmed that the best match to the experimental data is in region C (Fig. 3h). This suggests that the high fluidity of E8.5 epithelium is achieved in the regime of high junctional tension and contractility characteristic of region C. This is consistent with observations that the maintenance of high junctional tension is needed for proper neural tube closure at early developmental stages^29,30^.

## IKNM fluidizes the neuroepithelium

**Fig. 4:**
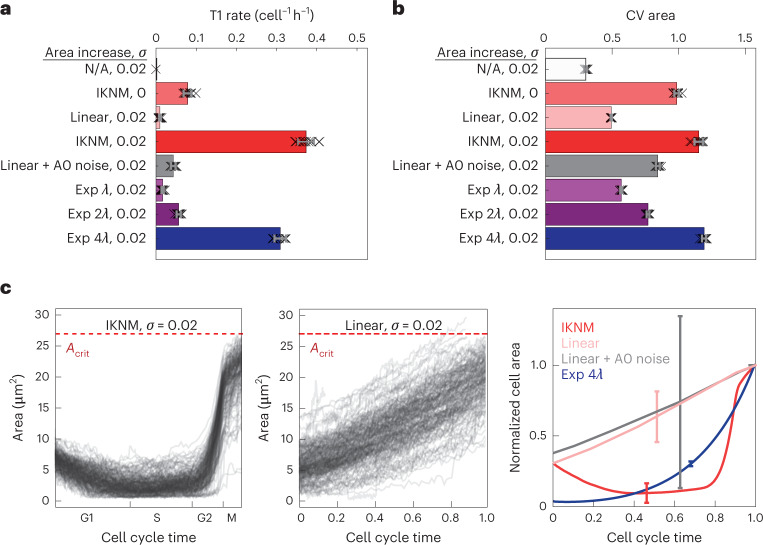
Tissue fluidization at high contractility/tension by cell cycle-dependent cell area dynamics. **a**,**b**, T1 rate (**a**) and mean cell area CV (**b**) for simulations with different modes of cell area increase during the cell cycle and different levels of noise (*σ*) as indicated. N/A indicates no division; IKNM, cell cycle-dependent target area; linear, linear area increase; linear + A0 noise, linear area increase with A0 noise; exp *nλ*, exponential increase with rate *nλ*, where *n* is indicated (Methods). Error bars, standard deviation from ten simulations per condition. Region C (${{{\bar{\varLambda }}}}$ = −0.074, ${{{\bar{\varGamma}}}}$ = 0.12). Double-sided *t-*tests for all conditions compared with the default: *P* < 0.0001. **c**, Apical area of 200 randomly selected individual cells in simulations with conditions: IKNM with *σ* = 0.02 (left); linear with *σ* = 0.02 (middle). The mean cell area during the cell cycle (normalized to the maximum) is shown in **c** (right) for the indicated conditions with *σ* = 0.02; sample sizes (number of cells), IKNM (*n* = 4,625); linear (*n* = 3,880); linear + A0 noise (*n* = 3,984); exp 4*λ* (*n* = 12,725). The cell cycle time is normalized from cell birth to cell division (end of mitosis). Error bars, standard deviation. Source data

High fluidity in region C has not been previously observed; hence, we investigated how the high level of cell rearrangements in this region arises. Because the implementation of an IKNM effect and ${{{\bar{\varLambda}}}}$ noise are distinct features of our model, we first compared how the rate of T1 transitions depends on these features (Fig. 4a). In the absence of any cell divisions and noise, T1 transitions are not observed. In the absence of cell divisions, in the presence of only junctional noise with *σ* = 0.02, the rate of T1 transitions in region C was also zero, similar to what is expected from the solid ground state of the network in this parameter region. The implementation of cell division by IKNM without ${{{\bar{\varLambda }}}}$ noise resulted in a low T1 rate (<0.1 cell^−1^ h^−1^). A classical implementation of cell divisions without an IKMN effect, but with linear cell area increase during the cell cycle, both with or without ${{{\bar{\varLambda}}}}$ noise, also resulted in a low T1 rate (<0.1 cell^−1^ h^−1^) (Fig. 4a and Supplementary Fig. 3a,b). By contrast, the IKNM effect and ${{{\bar{\varLambda}}}}$ noise together increased the T1 rate to 0.37 ± 0.02 cell^−1^h^−1^ and resulted in levels of clone fragmentation that are comparable with the experimentally observed value. This indicates that junctional noise on a timescale of minutes and fluctuations induced by IKNM (on a longer timescale of minutes to hours) cooperate to induce an increase in T1 rates that effectively fluidizes the tissue.

**Extended Data Fig. 3:**
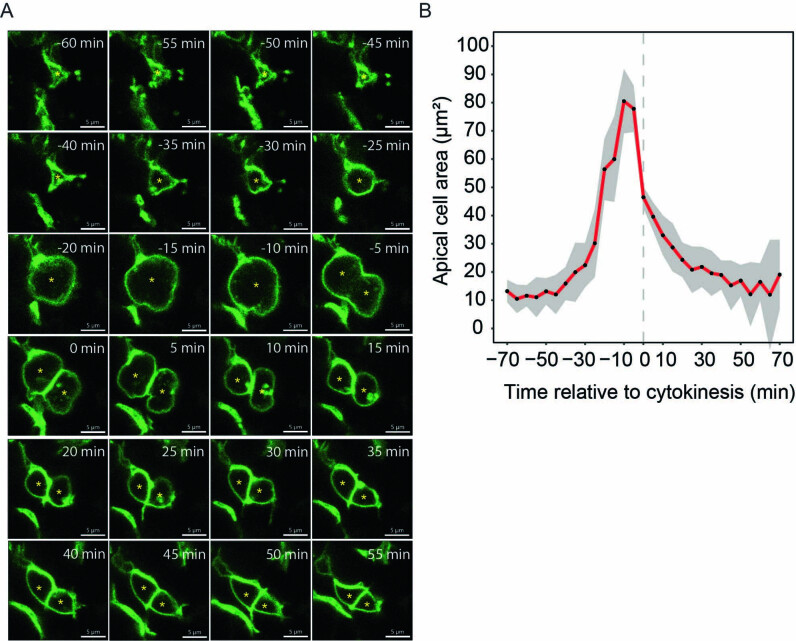
Cell area of neural progenitors at cell division. **A**. Snapshots from confocal time lapse imaging (Supplementary Video 5) of neuroepithelial cells expressing membrane GFP at E10.5. The first subapical z-section (shown) was used for tracing and quantification. Asterisk marks a dividing cell and its daughters. Scale bars, 5 μm. **B**. Mean cell area over time quantified from time-lapse movies such as the one shown in A. n = 17 cells from 3 embryos, shaded regions 95% confidence intervals. Time = 0 is the first time point where two distinct daughter cells were detected. Dividing cells rapidly increase their apical area prior to mitosis. Source data

To further explore how IKNM is associated with T1 transitions, we analysed the quartets of adjoining cells undergoing T1 transitions in simulations. This revealed that T1 quartets have a distinct distribution of mean cell areas compared with random quartets of cells (Supplementary Fig. 3c). In particular, T1 quartets have, on average, one large cell and three smaller cells. Consistent with this distribution, a fraction of T1 transitions were followed by cell division of the largest cell in the simulations (Supplementary Fig. 3d). Nevertheless, the majority of T1 events did not coincide with cell divisions and could be either preceded or followed by cell divisions (Supplementary Fig. 3e). Consistent with this, we observed examples of T1 transitions occurring before cell division, after cell division, coincident with division or in the absence of cell division in short-term live-imaging experiments of mouse embryos expressing ZO1–GFP (Supplementary Fig. 4a–d). Furthermore, cell divisions that we observed in time-lapse imaging of neural epithelia mosaically expressing membrane-localized GFP (*n* = 17 dividing cells; Extended Data Fig. 3) were not associated with the separation or rearrangement of daughter cells within at least 30 min after cytokinesis. In addition, treatment with calyculin A, which leads to basolateral enrichment of F-actin (Supplementary Fig. 4e) and has been shown to increase junctional stability at mitosis and prevent direct daughter-cell separation on cytokinesis^11^ (Methods), does not affect clone fragmentation in the neural tube (Supplementary Fig. 4f). Altogether, these observations suggest that cell rearrangements are not driven by the mitotic cell or its daughter cells in a direct cell autonomous manner.

To further investigate how IKNM influences cell rearrangements, we asked if the high cell area heterogeneity in the presence of IKNM (Fig. 4b) is sufficient to account for the increased cell rearrangements in region C. To address this possibility, we simulated a tissue without IKNM, in which the cell area grows linearly during the cell cycle, but with target cell areas drawn from a random distribution with CV comparable with the experimentally measured one (linear + A0 noise condition (Methods)). These simulations show that increasing the target cell area heterogeneity is not sufficient to increase the rate of T1 transitions (Fig. 4b,c). An alternative possibility is that the specific kinetics of cell area increase during the cell cycle generated by IKNM leads to a higher rate of T1 transitions. Consistent with this idea, the apical target area that increases exponentially over the cell cycle can generate increased T1 transitions. Furthermore, the sharper the increase in exponential growth rate, the higher is the area heterogeneity and higher is the rate of T1 transitions (Fig. 4a,b). Altogether, this analysis suggests that the specific cell area dynamics during the cell cycle, that is, the sustained low cell area during interphase and rapid increase at mitosis, are crucial for cell rearrangements in region C.

Consistent with the model, our experimental data from EdU- and pH3-labelling experiments show that cells in G2 have similar apical areas to cells in the S phase, but lower than cells in mitosis (Fig. 2c). This argues against a linear increase in cell area during the cell cycle and suggests that the apical cell area rapidly increases during cell division. Time-lapse imaging of neural epithelia expressing membrane GFP confirmed that the subapical cell area increases several times within less than 60 min before cytokinesis (Extended Data Fig. 3). These kinetics are similar to the rapid increase in apical area observed before cell division in simulations (Fig. 4c). Altogether, these data support the results of the model and indicate that the kinetics of cell area variability that fluidizes the neuroepithelium is driven by the cell cycle and reflects the changing apicobasal nucleus position during IKNM.

## Cell cycle dynamics influence cell rearrangements

**Fig. 5:**
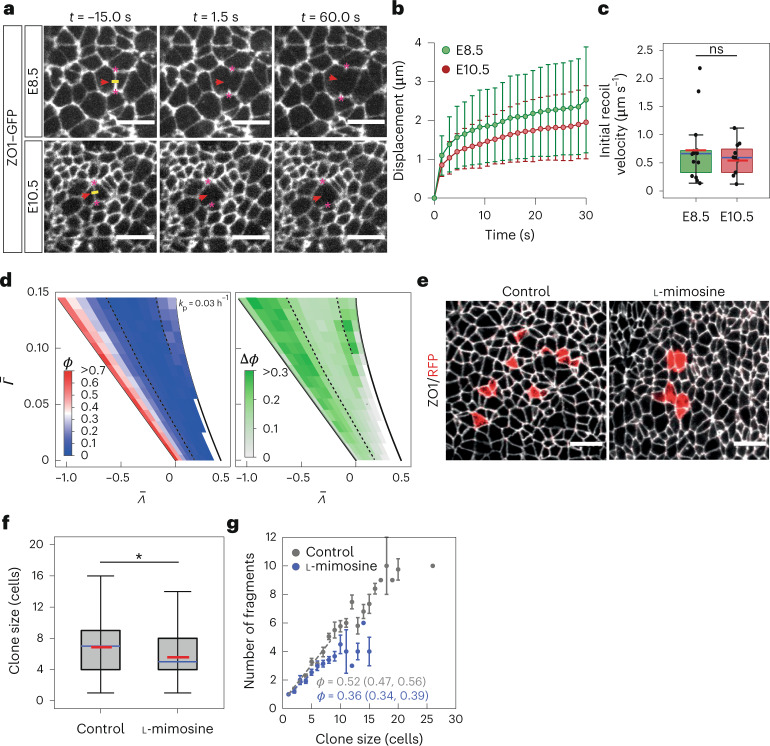
Extent of cell rearrangements depends on proliferation rate. **a**, Laser ablation of apical junctions at E8.5 and E10.5. Apical view, anterior left, dorsal up. The laser cut was performed at *t* = 0 s along the yellow line. The vertex positions (asterisks) were tracked to measure the recoil velocity. Scale bars, 10 μm. **b**, Mean displacement of the vertices over time. Error bars, 95% CI. **c**, Initial recoil velocity of vertices after laser ablation at the indicated stages (Methods). Mann–Whitney test; *P* > 0.05 (with and without outliers). Samples sizes in **b** and **c** are as follows: E8.5 (*n* = 14 ablations); E10.5 (*n* = 11 ablations) (Supplementary Table 1). 25–75 percentile (box), median (blue), mean (red), highest/lowest observations without outliers (whiskers). **d**, Left: fragmentation coefficient (*ϕ*) for simulations with low proliferation rate *k*_p_ = 0.03 h^−1^. Right: difference in *ϕ* between high (0.09 h^−1^; Fig. 3a) and low (0.03 h^−1^) *k*_p_. **e**, Confetti clones (red) induced at E7.5, embryos cultured from E8.5 for 42 h with vehicle or 210 μM l-mimosine. ZO1 immunostaining, white. Scale bars, 10 μm. **f**, Clone size quantification for **e**. Mann–Whitney test (two sided); *P = 0.019. Box plots as in **c**. Sample sizes are as follows: control (*n* = 382 clones); l-mimosine (*n* = 155 clones). **g**, Mean number of fragments per clone for the experiment in **e** and **f**. Corresponding fragmentation coefficient *ϕ* (95% CI) was obtained using a linear fit to the data for clones ≤8 cells (dashed lines). Error bars, s.e.m.; sample size as shown in **f**. Source data

Despite the presence of IKNM throughout development, the extent of clone fragmentation declines after E8.5, which raises the question of how this change is regulated. One possibility is that the mechanical parameters (${{{\bar{\varLambda}}}}$ and/or ${{{\bar{\varGamma}}}}$) change over time, such that the tissue ends up in the solid-like region B at later stages. To test this possibility, we performed laser ablation of individual cell junctions in E8.5 and E10.5 neural tubes which expressed ZO1–GFP (Fig. 5a,b, Supplementary Videos 6 and 7 and Methods). We observed no significant difference in the initial recoil velocity of vertices following laser ablation between the two developmental stages, suggesting that the active tension at these stages is similar (Fig. 5c). Further supporting this conclusion, an analysis of the cell shapes in neuroepithelia from E9.5, E10.5 and E11.5 embryos revealed that the experimentally observed cell shapes are consistent with parameter values characteristic of region C (Supplementary Fig. 5). These results suggest that changes in ${{{\bar{\varLambda}}}}$ and ${{{\bar{\varGamma}}}}$ are not the major factors underlying the change in tissue fluidity over time.

The impact of IKNM on clone fragmentation revealed by our model suggests that the cell division rate could be critical for regulating the extent of cell rearrangements by controlling the level of active stresses that generate fluctuations in the tissue. Between E8.5 and E10.5 of development, the proliferation rate decreases and terminal differentiation commences^18^, which lowers the net tissue growth rate by about twofold (Supplementary Fig. 1d). To test whether this could lead to tissue solidification, we lowered the proliferation rate in the vertex model simulations from 0.09 to 0.03 h^−1^. This resulted in a strong decline in the fragmentation coefficient of clones throughout most of the (${{{\bar{\varLambda}}}}$,${{{\bar{\varGamma}}}}$) parameter space (Fig. 5d and Supplementary Fig. 6a). In region C, *ϕ* declined by about twofold and a corresponding decline in T1 rates was observed (Supplementary Fig. 6b), whereas the cell area CV was reduced to a lesser extent and remained significantly higher than in region B (Supplementary Fig. 6c). This reduction in fragmentation coefficient in the model is reminiscent of the experimentally observed reduction in *ϕ* (Fig. 1e), suggesting that the decreasing rate of proliferation over time is a key driver of the decline in cell rearrangements.

This analysis predicts that artificially lowering the proliferation rate would lead to a lower level of cell rearrangements. To test this, we induced Confetti clones at E7.5 and then cultured the embryos from E8.5 in the presence of cell cycle inhibitors l-mimosine or aphidicolin for 42 h (Fig. 5e and Supplementary Fig. 7a). As expected, these treatments resulted in reduced mean clone sizes compared with control embryos (Fig. 5f and Supplementary Fig. 7b). Crucially, a comparison of the inhibitor-treated with vehicle-treated control embryos showed that for a given clone size, the number of fragments per clone was significantly reduced in both l-mimosine- and aphidicolin-treated conditions (Fig. 5g and Supplementary Fig. 7c). These results are in agreement with the model prediction and confirm that the proliferation rate has a profound influence on the extent of cell rearrangements in the neuroepithelium.

Besides the rate of proliferation, the overall rate of tissue growth can also be affected by cell loss. From E9.5 to E10.5 of development, terminal differentiation in the pMN domain results in the loss of progenitors from the neuroepithelium and also contributes to lowering the growth rate in this domain^18^. To test the effect of progenitor cell loss by terminal differentiation on cell rearrangements, we modelled cell loss in silico by randomly assigning a zero target area to a fraction of cells (Methods). These simulations showed that cell loss leads to a decrease in clone fragmentation throughout the (${{{\bar{\varLambda }}}}$,${{{\bar{\varGamma }}}}$) parameter space (Supplementary Fig. 7d). Consistent with this prediction, Confetti clones in the pMN domain have significantly lower fragmentation coefficients than clones in the pD domain, where the differentiation rate is lower in this time interval (Supplementary Fig. 7e). This indicates that in addition to decreasing the proliferation, the increasing terminal differentiation rate also contributes to reduce the extent of cell rearrangements in the neural epithelium over developmental time. Altogether, these observations strongly suggest that the increasing solidification of the mouse neuroepithelium over time is controlled by the observed changes in cell cycle dynamics over time.

Changes in the tissue growth rate have previously been linked to alterations in anisotropic growth and tissue morphogenesis^21,31^. To understand the consequences of reduced proliferation and therefore cell rearrangements for tissue morphogenesis, we treated E8.5 embryos with aphidicolin for 8 h. This resulted in striking changes in the shape of the neural plate, where the treated embryos had an increased ratio of anterior–posterior to dorsoventral length of the neural plate (Supplementary Fig. 8). This result is consistent with previous predictions of our model^21^ and indicates that there is an inherent link among tissue fluidity, growth rate and tissue shape.

## Conclusion

Morphogenetic processes have been recently linked to transitions in the material properties of tissues^32^. Here we demonstrate that in the mouse neural tube epithelium, there is a significant decline in tissue fluidity around E9.5 of development. Our data suggest that this decline resembles a glass transition, controlled by changes in active stresses within the tissue. We show that in the neuroepithelium, active stresses are generated by IKNM during the cell cycle. Consequently, the proliferation rate determines the extent of cell rearrangement and tissue fluidity.

Previous studies have shown that tissue rheology can change in the absence of noise or fluctuations^3,4,33,34^. In many cases, transitions in fluidity have been linked to changes in cell density or in mechanical properties, such as cell adhesion, cortical tension and contractility^2,3,5,6,33,35,36^. Tissue fluidity can also decline as a result of T1 delay times or nonlinear junction behaviours^37–39^. By contrast, the role of cell divisions, apart from a few experimental examples^3,10,11^ and theoretical predictions^9,40–43^, has been largely underappreciated. Slowing down of growth is a hallmark of development and has been measured in multiple tissues^44–46^. Our findings, therefore, suggest that increases in tissue rigidity over developmental time could be a natural consequence of the cell cycle dynamics in many tissues.

IKNM is the characteristic of many epithelia^47–49^, and hence, our finding that IKNM fluidizes the epithelia might be relevant to other tissues besides the neural tube. Our analysis indicated that IKNMs exert their effect on cell rearrangements by affecting the apical surface-area kinetics of cells, which results in large cell area variation. Interestingly, in the *Drosophila* wing disc, the presence of smaller-than-normal mutant cells has been shown to induce clonal fragmentation^19^. Yet, in our analysis, imposing ‘static’ cell area variation did not lead to high clonal fragmentation, indicating a distinct mechanism where the kinetics of cell area changes are crucial for epithelial rearrangements.

## Methods

### Experiments

#### Mouse strains and generation of clones

All the animal procedures were performed in accordance with the relevant regulations and were approved under the license BMWFW-66.018/0006-WF/V/3b/2016 from the Austrian Bundesministerium für Wissenschaft, Forschung und Wirtschaft. The following strains were previously described: MADM-11^*TG*^ and MADM-11^*GT*^ (ref. ^17^), Rosa26–Confetti (Brainbow-2.1 (ref. ^50^)), Sox2–CreERT2 (ref. ^51^), mTmG (ref. ^52^), R26–ZO1–GFP (ref. ^53^), Rosa26–tdTomato (ref. ^54^). To generate MADM clones in MADM^*TG/GT*^ trans-heterozygous Sox2–CreERT2-expressing embryos, MADM-11^*TG/TG*^ mice were bred to MADM-11^*GT/GT*^ and Sox2–CreERT2/+, and pregnant females were injected with 3 mg per mouse of tamoxifen. To generate Confetti clones, heterozygous Sox2–CreERT2 mice were bred to heterozygous Rosa26–Confetti and pregnant mothers were injected with 0.75 mg per mouse of tamoxifen. Tamoxifen stock was prepared fresh in sunflower oil.

The first time point where we observe labelled cells is 8 h after tamoxifen injection (Supplementary Fig. 1b), reflecting the time it takes for the nuclear translocation of Cre and subsequent onset of reporter expression. Thus, the time of Cre activity in the 24 h tracing experiments (Fig. 1) is considered to be 16 h.

#### Immunohistochemistry, EdU incorporation and imaging

For E9.5 and later stages, embryos were bisected along the roof plate before fixation and along the floor plate before immunostaining. Embryos were fixed in 4% paraformaldehyde and subsequently in methanol. Primary and secondary antibody incubations were 24 h each. Washes in phosphate-buffered saline with 0.1% Tween were 10 h each. The brachial region was flat mounted with grease spacers between slide and coverslip. Primary antibodies used were mouse anti-ZO1 (Invitrogen, 1:90), goat anti-Olig2 (R&D Systems, 1:100), sheep anti-GFP (AbD Serotec, 1:1,000), rabbit anti-RFP (Rockland, 1:2,000), mouse anti-Nkx2.2 (DSHB, 1:20), rat anti-pH3 (Sigma,1:1,000), goat anti-SOX2 (R&D Systems, 1:100), rabbit anti-Brachyury (Abcam, 1:100). Secondary antibodies used were donkey anti-mouse Alexa Fluor 647 and donkey anti-goat FITC (Jackson Immuno, 1:250), donkey anti-rabbit Cy3 and donkey anti-rat Cy3 (Jackson Immuno, 1:1,000), donkey anti-sheep FITC (Jackson Immuno, 1:250).

For MADM clone analysis, embryos were immunostained against ZO1, RFP, GFP and Nkx2.2. Clones located within 25 μm dorsal to the Nkx2.2 domain boundary were considered to be pMN clones. For Confetti clone analysis, embryos were immunostained against ZO1 and Olig2. To stain actin filaments, the following steps of the protocol were modified: embryos were fixed in 4% paraformaldehyde overnight, methanol fixation was omitted and Alexa Fluor 488 Phalloidin (Thermo Fisher Scientific, 1:100) was added together with the secondary antibody.

For EdU-labelling experiments, Sox2–CreERT2 mice were bred to ROSA26–tdTomato. Pregnant mice were intraperitoneally injected with 3 mg tamoxifen at E6.5 and with 0.5 mg EdU in phosphate-buffered saline (stock, 2.5 mg ml^–1^) on the day of the experiment. For S-phase labelling, mice were sacrificed 20 min after EdU injection at E8.5 and 30 min after injection at E10.5. To label cells in G2, mice were sacrificed 2 h after EdU injection. The embryos were dissected, fixed and immunostained against ZO1 and RFP, as described above. Subsequently, the incorporated EdU was detected using the Alexa Fluor 488 Click-iT EdU imaging kit and protocol (Invitrogen, C10337).

Imaging was performed using a 40×/1.3 numerical aperture oil objective on an LSM880 inverted confocal microscope. Images of the apical surface capturing the entire dorsoventral length of the epithelium were acquired through tile scanning with *Z* slices 0.8 μm apart. The tiles were configured in the form of a grid and overlapped 10%. Subsequently, the tiles were stitched using the BigStitcher plugin in Fiji version 2.9 (ref. ^55^).

#### Mouse embryo culture and inhibitor treatments

To combine clone tracing with mouse embryo culture and inhibitor treatments, heterozygous Sox2–CreERT2 mice were bred to Rosa26–Confetti. To induce sparse labelling (see the ‘Clone identification and fragmentation coefficient estimation’ section), pregnant mothers were injected with 0.75 mg per mouse of tamoxifen at E7.5. After 24 h, at E8.5, embryos were dissected and cultured with their yolk sac intact in temperature-controlled roller culture^56^ (5% CO_2_ and 20% O_2_). The embryo culture medium consisted of 1:1 rat serum: dissection medium^57^ (Gibco DMEM/F12 without phenol red (Thermo Fisher), 10% Gibco foetal bovine serum (Thermo Fisher), 1× penicillin–streptomycin (Sigma)). To perturb proliferation, embryos were cultured in the presence of 210 μM l-mimosine (Sigma) or 800 nM aphidicolin (Sigma) for 42 h. Calyculin A (Merck Millipore) was used at a final concentration of 0.6 nM for 42 h of culture. After culture, the embryos were harvested and processed for imaging, as described above.

#### Laser ablation

Embryos heterozygous or homozygous for R26–ZO1–GFP were collected at E8.5 and E10.5 of development. To perform laser ablation, whole E8.5 embryos or dissected flat-mounted E10.5 brachial neural tubes were immobilized for live imaging in glass-bottom dishes (Ibidi) in embryo culture medium (see ‘Mouse embryo culture and inhibitor treatments’ section) supplemented with 8 mg ml^–1^ fibrinogen (Millipore). Thrombin (0.5 U μl^–1^, Sigma Aldrich) was added to form a fibrin gel^18^. The samples were kept in an environmental chamber with 5% CO_2_ at 37 °C. Laser ablation was performed on an Andor spinning-disc system with inverted Axio Observer Z1, C-Apochromat 63×/1.2 water-immersion objective (Carl Zeiss) using a 355 nm pulsed UV-A nanolaser (Teem Photonics) at 1.8–1.9% laser power with 25 pulses (2 shots µm^–1^) at 1,000 Hz. Junction ablation was performed approximately at the centre of the cell edge between two vertices along a 2 µm line oriented along the A–P axis. Edges oriented dorsoventrally and located at intermediate dorsoventral positions were selected for the experiment. Images were collected with 250 ms exposure time and frame rate of 1.5 s. To determine the recoil velocities, the positions of the vertices were manually tracked over time in Fiji. The initial recoil velocity was defined as the distance between the vertices at t1 minus the distance at t0 (before the cut), divided by the time interval between t0 and t1 (1.5 s).

#### Live imaging

To image the apical surface of the neuroepithelium at the level of tight junctions, embryos heterozygous or homozygous for ZO1–EGFP were collected at E8.5 and E10.5. Whole E8.5 embryos and bisected E10.5 brachial neural-tube regions were positioned on 35 mm glass-bottom dishes (Ibidi) in an embryo culture medium (see the ‘Mouse embryo culture and inhibitor treatments’ section) and immobilized with coverslips on silicon grease spacers. Imaging of the ZO1–GFP-labelled apical surfaces was performed on an inverted LSM800 confocal microscope using a 40×/1.2× water objective. The *Z* stacks were acquired with *Z* slices 0.75 μm apart for a period of about 1–2 h.

Imaging of the cell membranes (marked by membrane GFP) at the subapical level was performed using E10.5 Sox2–CreERT2/+, mTmG/+ embryos of mothers injected with 1 mg tamoxifen 24 h before dissection. Brachial neural-tube regions were dissected and prepared for imaging the same way as that for laser ablation experiments. The *Z* stacks were acquired with *Z* slices 0.7 μm apart.

### Data analysis

#### Clone identification and fragmentation coefficient estimation

Images were processed in Fiji. Labelled progenitor cells were manually marked at their apical surface at the level of ZO1 staining. Fragments were defined as groups of adjacent cells that share an edge or a vertex. Clones were defined as groups of labelled progenitor cells in close proximity of each other (<25 μm to the nearest labelled cell).

In the case of MADM clones, G2 recombination followed by the X segregation of chromosomes in mitosis produces a GFP- and RFP-expressing daughter cell (Fig. 1a). Both GFP- and RFP-expressing cells were included in defining a clone. G2–Z segregation produces an unlabelled and a GFP/RFP-coexpressing daughter cell. G1 recombination produces a GFP/RFP-coexpressing cell. In our dataset, we found an increasing proportion of GFP/RFP-coexpressing cells over time (Supplementary Fig. 1a), which correlates with the increasing relative G1 duration over time^18^. This suggests that the majority of GFP/RFP-coexpressing clones result from G1 recombination. We, therefore, also included GFP/RFP-coexpressing clones in the analysis. MADM clones and the fragments they contain were manually identified from images.

In the case of Confetti clones, only the RFP, YFP and CFP reporters, which can be detected at the apical surface, were used for the analysis, whereas clones labelled by the nuclear GFP were excluded. The sparseness of labelling in the experiments was as follows: 322 ± 38, 366 ± 39 and 235 ± 36 cells mm^–2^ for CFP, RFP and YFP, respectively (mean ± standard error of the mean (s.e.m.) for 54 images is given). To identify Confetti clones, the cell coordinates were recorded and subsequently analysed using a custom-built Python script, similar to the one reported elsewhere^18^. CFP, RFP and YFP channels were separately analysed. The labelled cells were assigned to the same fragment if the distance between them was <5 μm and to the same clone if they were <25 μm apart. These assignments were consistent with the visual identification of fragments and clones, except in occasional cases where the clone size was unreasonably large. Labelled postmitotic neurons that have delaminated from the neural epithelium were excluded from the analysis.

We determined the fragmentation coefficient *ϕ* by fitting *f* = *ϕs* + *b* to the respective dataset, where *f* is the mean number of fragments for a given clone size, *s* is the size of the clone in cells and *b* is an offset parameter that is chosen in such a way that the line crosses through the point (1, 1), reflecting the fact that single-cell clones have one fragment by definition. For MADM clones analysed 24 h after tamoxifen injection, reliable statistics could be obtained for clones with four cells or less; hence, only these clone sizes were used for estimating *ϕ*. In mouse embryo culture experiments, Confetti clones were analysed 64 h after tamoxifen injection. In this case, reliable statistics could be obtained for clone sizes of ≤8 cells and these were used to estimate *ϕ*.

#### Growth rate estimation

The growth rate of MADM clones *k*_g_ was inferred from the mean clone size *s* as *k*_g_ = ln(*s*)/∆*t* , where ∆*t* = 16 h is the time interval of Cre activity in the experiments (Supplementary Fig. 1b–d and Methods).

#### Spread and anisotropy of clones

To estimate the spread of clones, the coordinates of cell centres in a clone were used to determine the clone centroid. The maximum spread of the clone was quantified as the distance between the clone centroid and furthest cell centre. To estimate the mean maximum spread for a given developmental stage, clones of all sizes were taken into account (including single-cell clones).

Clone anisotropy was quantified by drawing a bounding rectangle around the clone, using the apical cell outlines, marked by ZO1, to demarcate the cells. Images are always oriented so that the vertical axis is aligned with the tissue D–V axis. The aspect ratio of the clone is then given by the D–V to A–P side lengths of the bounding rectangle. Note that quantifying the clone shape at the apical surface, rather than the cell bodies or nuclei, avoids potential artefacts of tissue mounting, where the clone shape could be affected by the misalignment of the apical and basal surfaces of the neural epithelium.

To quantify the self-overlap function in simulations, we adapt the definition from other work^25,26^ for a growing tissue. The self-overlap function is defined as $Q_\mathrm{s}\left( t \right) = \frac{1}{N}\mathop {\sum }\nolimits_{k = 1}^N w\left( {\left| {\widetilde {r_k}\left( t \right) - \widetilde {r_k}\left( 0 \right)} \right|} \right)$, where $\widetilde {r_k}\left( t \right)$ is the position of the centre of mass of the *k*th cell at time *t*, *w* is a window function that gives 1 for $\left| {\widetilde {r_k}\left( t \right) - \widetilde {r_k}\left( 0 \right)} \right| \le \tilde R_{\mathrm{cell}}$ and 0 otherwise, and *N* is the number of cells. The $\tilde R_{\mathrm{cell}}$ value is the characteristic length that corresponds to the initial cell radius in the reference frame defined below. To correct for growth, we consider every cell trajectory in a reference frame that is centred at the initial position of that cell and normalized by the fastest growing dimension. In practice, we apply the following two steps: (1) for every newborn cell with position (*r*_D–V_, *r*_A–P_) at time *t* = 0, we shift the corresponding cell trajectory to start in (*r*_D–V_, *r*_A–P_)→(0, 0); (2) we then normalize the cell position over time by dividing its position by the D–V and A–P extensions of the growing tissue with the correction from the anisotropic growth, that is, $\widetilde {r_k}\left( t \right) = (\frac{{r_{\rm{D-V}}}}{{L_{\rm{D-V}}}},\frac{{r_{\rm{A-P}}}}{{L_{\rm{A-P}}}}\langle\frac{{L_{\rm{A-P}}}}{{L_{\rm{D-V}}}}\rangle)$, where *L*_D–V_ and *L*_A–P_ are the respective tissue dimensions at time *t*, and $\langle\frac{{L_{\rm{A-P}}}}{{L_{\rm{D-V}}}}\rangle$ is the average A–P/D–V ratio for a given simulation. The $\tilde R_{\mathrm{cell}}$ value is defined as $\frac{{\sqrt {\langle A\rangle }}}{2}/L_{\rm{D-V}}$, where 〈*A*〉 is the average cell area at time *t* = 0, and *L*_D–V_ is taken at *t* = 0.

#### Segmentation of cell shapes

Apical surfaces immunostained for ZO1 of the E8.5, E9.5, E10.5 and E11.5 neural tubes were segmented using the Tissue Analyzer plugin^58^ in Fiji. The cell outlines were automatically identified and manually checked for correctness. This plugin provided the description of a polygonal mesh including the vertices and edges of cell outlines as well as the number and identity of cell neighbours. The cell area was calculated using a standard formula for the area of the *n*-gon and cell perimeter as a sum of the length of polygon edges. The cell elongation was calculated as done elsewhere^21,27^.

In the EdU- and pH3-labelling experiments, the distance between the nucleus centre and ZO1-labelled junctional level was measured using Imaris 9.1 (Oxford Instruments) from confocal *Z* stacks. To associate EdU-labelled nuclei with the corresponding apical surfaces of cells, cells that expressed cytosolic tdTomato and therefore allowed tracing the cell body were used for the analysis.

Cell areas in the EdU experiment (Fig. 2) and membrane-GFP-expressing embryos in live imaging (Extended Data Fig. 3) were quantified using Imaris 9.1. For this, the cells were segmented using a watershed function. Subsequently, the cell area was estimated from the segmented cell volume within the relevant *Z* slice divided by the voxel depth. For the EdU experiments, the relevant *Z* slice is the one marked by the ZO1 expression. For the membrane-GFP-expressing embryos, the cell areas were quantified at the *Z* position corresponding to 2.1–2.8 μm below the apical cell membrane.

To quantify the variation in edge length over time, time-lapse images of ZO1–GFP-expressing neural tubes were segmented in Fiji in the same way as the fixed images. To avoid large fluctuations in edge length that arise as a result of cell divisions, T1 transitions or segmentation errors, we only quantified the edges that could be tracked throughout the duration of the experiment and were at least one cell away from the cells undergoing large fluctuations.

### Simulations

#### Vertex model description and implementation

The vertex model used in this study is based on another work^21^ and was implemented here using Python 3.7. Briefly, the following energy function is minimized in every simulation step:

1 $$E = \mathop {\sum }\limits_\alpha \frac{{K_\alpha }}{2}\left( {A_\alpha - A_\alpha ^0(t)} \right)^2 + \mathop {\sum }\limits_{ij} {{\Lambda }}_{ij}l_{ij} + \mathop {\sum }\limits_\alpha \frac{{{{\varGamma }}_\alpha }}{2}L_\alpha ^2$$

where *α* = 1,…, *N*_c_ enumerates all the cells; *i* = 1,…, *N*_v_ enumerates all the vertices; *K*_*α*_ is the elasticity coefficient; *A*_*α*_ is the area of cell *α*; $A_\alpha ^0(t)$ is the preferred area of cell *α* at time *t;* *Λ*_*ij*_ is the line-tension coefficient associated with the cell edge between *i* and *j* of length *l*_*ij*_; and *Γ*_*α*_ is the contractility coefficient of cell *α* with perimeter *L*_*α*_. We assume that the parameters are the same for each cell (*K*_*α*_ = *K*, *Γ*_*α*_ = *Γ*) and for each edge (*Λ*_*ij*_ = *Λ*) if no noise in the line tension is considered. The preferred cell area $A_\alpha ^0(t)$ is a piecewise linear function reflecting the effect of IKNM on the apical cell area in the four phases of the cell cycle (G1, S, G2 and M; also see below). Adopting the same notation as previous studies^21,22^, we use the normalized parameters as ${{{\bar{\mathrm \Lambda }}}} = \frac{{\Lambda }}{{K\left( {A^0} \right)^{3/2}}}$ and ${{{\bar{\mathrm \Gamma }}}} = \frac{{\Gamma }}{{KA^0}}$, where *A*^0^ is the average target area during the cell cycle.

The motion of vertices is determined from the first-order kinetics: $\frac{{\mathrm{d}r_i}}{{\mathrm{d}t}} = - \frac{1}{\mu }\frac{{\partial E}}{{\partial r_i}}$, where *r*_*i*_ is position of vertex *i* and *μ* is the drag coefficient. Tissue growth was considered to be anisotropic with drag coefficients *μ*′ and *μ*″ in the D–V and A–P directions, respectively^21^.

The following changes were made in the current version of the model:

##### Implementation of junctional noise

We considered that fluctuations in the internal line tension follow an Ornstein–Uhlenbeck process, namely, $\frac{{\mathrm{d}{{\Lambda }}_{ij}}}{{\mathrm{d}t}} = - \frac{1}{\tau }\left( {{{\Lambda }}_{ij} - {{\Lambda }}_{ij}^0} \right) + {{{{\xi }}}}_{{{{{ij}}}}}\left( t \right)$, where *ξ*_*ij*_(*t*) is white, uncorrelated noise with ‹*ξ*_*ij*_(*t*) = 0› and $\langle\xi _{ij}\left( t \right)\xi _{kl}\left( {t^\prime } \right) \rangle= \frac{{2\sigma ^2}}{\tau }\delta _{ik}\delta _{jl}\delta (t - t^\prime )$. We used the following discretization^23^:

2 $${{\Lambda }}_{ij}\left( {t + {{\Delta }}t} \right) = {{\Lambda }}_{ij}\left( t \right) - \frac{{{{\Delta }}t}}{\tau }\left( {{{\Lambda }}_{ij}\left( t \right) - {{\Lambda }}_{ij}^0} \right) + \sqrt {\frac{{2\sigma ^2{{\Delta }}t}}{\tau }} \overline {\xi _{ij}} (t)$$

where Δ*t* is a time step used in the simulation, *τ* is the line-tension correlation time, *σ* is the intrinsic line-tension deviation, $\Lambda_{ij}^{0}=\Lambda$   is a reference line tension that corresponds to the line tension without noise and $\overline {\xi _{ij}} \left( t \right)$ is drawn from the Gaussian distribution *N*(0, 1).

##### Implementation of T1 and T2 transitions

The T1 transition is defined elsewhere^21^. In particular, when an edge between two neighbouring cells is shorter than a predefined small length *l*_T1_, this edge is replaced with a new edge that is perpendicular to the old edge and has a length *l*_new_ = 1.01*l*_T1_. Using this definition, we observed that for negative line tension or in the presence of line-tension fluctuations, immediately after a T1 transition, the new edge can shrink instead of extending, thus leading to a reverted T1 transition. This can occur multiple times at a given edge, and hence, we call this an oscillatory T1 transition.

One strategy to partly mitigate the occurrence of oscillatory T1 transitions is to increase the *l*_new_/*l*_T1_ ratio^59^. However, particularly in region A, the oscillatory T1 transitions are generic and increasing *l*_new_/*l*_T1_ does not result in decreasing the number of oscillatory T1 transitions. Therefore, we approached this instead by keeping track of the oscillatory T1 transitions and subtracting them from the overall count of T1 events in our statistics. More specifically, we track how many T1 transitions occurred for every edge, using the dictionary data structure in Python. If repeated T1 transitions occurred *n*_T1_ times between time *t*_0_ and *t*_*n*T1_, their contribution to the T1 unique rate, namely, T1_UNQ_, was considered to be 1/*n*_T__1_ for times between *t*_0_ and *t*_*n*T1_.

T2 transitions are defined in another work^21^. In particular, cells in which the area becomes very small have shrinking edges. This results in sequential T1 transitions, which finally lead to a double-sided cell with zero area. Such cells are removed from the simulation by merging the two vertices that delimit the double-sided cell into one vertex. The last T1 transition that results in a double-sided cell is counted as a T2 transition, and is not included in the overall number of T1 transition events. All the T1, T1_UNQ_ and T2 rates are estimated in time windows of Δ*t* = 2 h by the dividing number of respective events with the average number of cells in this time window. The T1 rate reported in the main text as well as in Figs. 3b and 4a and Supplementary Fig. 6b is defined as the T1 unique rate.

Cell removal from the tissue through differentiation is implemented similar to another work^21^ with the additional requirement that if the cell was randomly selected for differentiation, the line-tension coefficients *Λ*_*ij*_ for this cell are no longer fluctuating and have assigned a positive value of *Λ*_*ij*_ = 0.2, which fosters shortening of all the edges of this cell.

##### Cell lineage tracing

To efficiently analyse in silico clonal populations, the complete information about cell lineage, that is, daughter-cell identifiers and division times, are stored. For the analysis of clone fragmentation in silico, we used all the clones per simulation and ten independent simulations per parameter set.

##### Parameters of the model

The used parameters are summarized in Supplementary Table 2. The default proliferation rate in the model *k*_p_ = 0.09 h^−1^ has been chosen to match with the experimentally observed tissue growth rate (Supplementary Fig. 1d): it results in mean sizes of simulated clones of three to four cells (depending on the exact value of ${{{\bar{\Lambda }}}}$ and ${{{\bar{\varGamma }}}}$), which is similar to the clone sizes observed in injection at E8.5 (Supplementary Fig. 1c). The specific proliferation (*k*_p_) and differentiation (*k*_n_) rates used in the simulations are given in the corresponding figure legends. The critical area *A*_C_ has been set to 27 µm^2^ so that the range of cell areas in the simulations (Supplementary Fig. 2a) is comparable with the range of areas measured in the experiments (Supplementary Fig. 5b). Furthermore, *A*_C_ = 27 µm^2^ results in a very close agreement between the mean edge length in simulations and in the E10.5 experimental data (1.47 ± 0.01 μm and 1.43 ± 0.01 μm (mean ± s.e.m.), respectively; Fig. 2d). The length of a simulation step Δ*t* = 0.29 s has been chosen such that the model has high temporal resolution and includes on the order of 10^5^ simulation points per cell cycle (10^5^ points correspond to 8 h). The units of force are arbitrary. Every data point across the (${{{\bar{\Lambda }}}}$,${{{\bar{\varGamma }}}}$) parameter space was obtained by pooling together cells from ten independent simulations for a given set of parameters $({{{\bar{\Lambda }}}},{{{\bar{\varGamma }}}},k_\mathrm{p},k_\mathrm{n})$.

##### Vertex model initialization and in silico clone tracing

The vertex model is initiated with a regular hexagonal lattice of ten rows with ten cells per row. In the initial simulation phase, the tissue grows for 16 h with ${{{\bar{\Lambda }}}}$ = −0.184, ${{{\bar{\varGamma }}}}$ = 0.07, *k*_p_ = 0.09 h^−1^, *k*_n_ = 0 h^−1^ and *σ* = 0. In our default simulations, we used distinct drag viscosities for the D–V and A–P dimensions, resulting in rectangular tissues with A–P/D–V length ratios of <1 at the end of the simulations (Supplementary Videos 2–4). After the initial simulation phase, the number of cells is 460 ± 19 (mean ± standard error), the time is set to 0 and the parameters are modified to the target simulation parameters. The tissue is then allowed to grow with the target simulation parameters for 8 h. Subsequently, clones are labelled and clonal populations of cells are tracked for 16 h.

#### Analysis of cell area heterogeneity and kinetics

To investigate the area variability of cells undergoing a T1 transition, we defined quadruplets of neighbouring cells, designated as A, B, C and D, where A and B share a common edge and C and D do not (Supplementary Fig. 4c). We further define the cell names based on the cell area such that area of A < area of B and area of C < area of D. If the common edge between A and B shrinks below *l*_T1_, a T1 transition takes place, as a result of which A and B are no longer adjacent, whereas C and D become new neighbours. For comparison, ‘random’ quadruplets are generated by randomly finding A and B cells separated by a common edge, and finding cells C and D that are adjacent to A and B, but not to each other. Note that because the polygonal mesh has no rosettes, that is, each vertex has three edges associated with it, the assignment of a quadruplet to an edge is unique.

##### Cell area kinetics during cell cycle and cell division

The IKNM is approximated as a linear combination of two terms, one corresponding to a linear increase in cell volume and the other interpolating for the change in apical cell surface as a function of the age of a cell:

3 $$A_\alpha ^0\left( t \right) = \frac{1}{2}(g_\alpha {{\Delta }}t + 1)(\rho _\alpha \left( {{{\Delta }}t} \right)^2 + 1)$$

where *g*_*α*_ is the growth rate of the cell *α*, Δ*t* = *t* – *t*_new_ is the age of the cell that divided at *t*_new_ and *ρ*_*α*_(Δ*t*) is a piecewise linear function representing the apical–basal position of the nucleus as a function of Δ*t* and cell cycle phase^21^. This function equals to zero in the S phase of the cell cycle in which the nucleus stays basal, and its value is 1 during mitosis when the nucleus is apical. The exact form of *ρ*_*α*_(Δ*t*) is defined elsewhere^21^. The growth rate *g*_*α*_ is drawn from a normal distribution with mean equal to 1/*t*_T_, where *t*_T_ is the total cell cycle time, and standard deviation *σ*_g_ = 0.45/*t*_T_. Negative growth rates are not allowed. In the simulation, the proliferation is defined as *λ* = ln(2)/*t*_T_. The cell divides when the cell is in the M phase, that is, $\frac{{{{\Delta }}t}}{{t_\mathrm{T}}}$ > 0.9, and the cell volume exceeds a critical value, namely, $A_\alpha ^0\left( t \right)$ > *A*_C_. The cell divides by introducing a new edge that splits this cell into two daughter cells that enter the next cell cycle^21^.

To study how the cell area kinetics affects T1 transitions, we use different forms of $A_\alpha ^0\left( t \right)$ and different levels of line-tension noise *σ*. In Fig. 4, different conditions for area kinetics are defined as follows:

‘N/A’, no division, *σ* = 0.02

‘IKNM’, $A_\alpha ^0\left( t \right)$ as in equation (3), *σ* = 0;

‘Linear’, cell divisions without IKNM (that is, *ρ*_*α*_(Δ*t*) = 1 in equation (3)), *σ* = 0.02;

‘IKNM’, default condition with $A_\alpha ^0\left( t \right)$ as in equation (3) and ${{{\bar{\mathrm \Lambda }}}}$ noise as in equation (2) with *τ* = 37 s, *σ* = 0.02;

‘Linear + A0 noise’, apical–basal position of nucleus in equation (3) is replaced with *ρ*_*α*_(Δ*t*) = *z*, where *z* is drawn from a uniform distribution ranging from 0 to 2; *σ* = 0.02;

‘exp *λ*’, ‘exp 2*λ*’, ‘exp 4*λ*’, a linear increase in cell area is replaced with an exponential increase, that is, $A_\alpha ^0\left( t \right) = \frac{1}{2}({{{\mathrm{exp}}}}(g_\alpha {{\Delta }}t) + 1)$; *λ*, 2*λ* and 4*λ* correspond to *g*_*α*_ = 1, 2 and 4, respectively; *σ* = 0.02.

#### Comparison between model and data

To estimate *σ* from the experimental data, simulations were adjusted to generate 48 frames every 25 s, so that the timescale is comparable with the live-imaging experiment and similar to the correlation time *τ* = 37 s. Simulations were initialized for 16 h with initialization parameters followed by 8 h with target parameters and defined magnitude of noise (*σ* = 0, 0.01 and 0.02). After that, the edges were tracked and filtered to avoid segmentation errors or fluctuations due to divisions and T1 transitions influencing the outcome. In particular, the edges were selected only if the two cells connected by an edge did not divide or undergo a T1 transition for the tracking interval of 48 frames. Furthermore, only the tracked edges that shared their two vertices with other tracked edges were analysed.

For every edge, the relative edge length is defined as $\tilde l=l/\bar l$, where *l* is the edge length at a particular time and $\bar l$ is the mean edge length throughout the time interval. The standard deviation of the relative edge lengths ${{{\mathrm{std}}}}(l/\bar l)$ over time changes with the absolute edge length (Fig. 2d) in a manner that depends on the value of *σ*. This was used to compare the simulations with the experimental data for different *σ* values.

To compare the cell shapes between vertex model simulations and experimental data, we use the cumulative distance between the model and experimental data including the following non-dimensional descriptors *D* ∈ {*p*_0_, *ϵ*, *α*, *hex*, *p*_0CV_, *ϵ*_CV_, *A*_CV_, *P*_CV_} (Supplementary Table 3). For each descriptor *D*, we calculate a difference $D_{{\Delta }} = \left| {\overline {D_{\mathrm{exp}}} - \overline {D_{\mathrm{sim}}} } \right|/\widehat {\sigma _D},$, where ${\overline {D_{\mathrm{exp}}}}$ is the mean value of *D* obtained for all the cells in the simulation for a given set of parameters $({{{\bar{\Lambda }}}},{{{\bar{\varGamma }}}},k_\mathrm{p},k_\mathrm{n})$ at final time, $\overline {D_{\mathrm{sim}}}$ is the mean value of *D* estimated for the segmented data at a specific developmental stage and $\widehat {\sigma _D}$ is the standard deviation of $\left| {\overline {D_{\mathrm{exp}}} - \overline {D_{\mathrm{sim}}} } \right|$ over all the samples. The cumulative distance *Δ*_tot_/*n* is then defined as the sum of differences *D*_Δ_ for all the descriptors, normalized to the number of descriptors, that is, $\frac{{{{\varDelta }}_{\mathrm{tot}}}}{n} = \frac{1}{{{{{\mathrm{number}}}}\;{{{\mathrm{of}}}}\;{{{\mathrm{descriptors}}}}}}\mathop {\sum }\limits_D D_{{\Delta }}$.

The *z* score for a given cell shape descriptor is defined as $z = \left( {\overline {D_{\mathrm{sim}}} - \overline {D_{\mathrm{exp}}} } \right)/\sigma _{\overline {D_{\mathrm{exp}}} }$, where $\sigma _{\overline {D_{\mathrm{exp}}} }$ is the standard deviation of the mean $\overline {D_{\mathrm{exp}}}$ estimated for different experimental images (Supplementary Table 1). In Extended Data Fig. 2, we report the absolute value of *z* score.

### Software and code

The vertex model code used in this study is available via GitHub at https://github.com/mpzagorski/vertex_model_python_3. The code is modified from another work^21^ to include an adaptation to Python 3.7, implementation of junctional noise, handling oscillatory T1 transitions and cell lineage tracing (Methods). Custom code in Mathematica 12.1 (Wolfram) was used to analyse the results of the vertex model, estimate fragmentation coefficient, cell shape descriptors, and comparison between simulation and experimental data. Supplementary Videos 2–4 are generated using custom Mathematica 12.1 code by post-processing the results of the vertex model simulation.

### Reporting summary

Further information on research design is available in the Nature Portfolio Reporting Summary linked to this article.

## Online content

Any methods, additional references, Nature Portfolio reporting summaries, source data, extended data, supplementary information, acknowledgements, peer review information; details of author contributions and competing interests; and statements of data and code availability are available at 10.1038/s41567-023-01977-w.

## Supplementary information

Supplementary Information Supplementary Figs. 1–8 and Tables 1–3.

Reporting Summary

Supplementary Video 1 Time-lapse imaging of ZO1–GFP-expressing neural tube at E8.5. Cells were segmented and bonds were tracked to estimate the variation in edge length over time (Fig. 2d).

Supplementary Video 2Simulations performed in parameter region A ($\bar{\varGamma}$ = 0.12 and ${{{\bar{\Lambda }}}}$ = −0.711). Example clones are displayed in different colours. *k*_p_ = 0.09 h^−1^, *k*_n_ = 0 h^−1^.

Supplementary Video 3Simulations performed in parameter region B (${{{\bar{\mathrm \Gamma }}}}$ = 0.12 and ${{{\bar{\Lambda }}}}$ = −0.393). Example clones are displayed in different colours. *k*_p_ = 0.09 h^−1^, *k*_n_ = 0 h^-1^.

Supplementary Video 4Simulations performed in parameter region C (${{{\bar{\mathrm \Gamma }}}}$ = 0.12 and ${{{\bar{\Lambda }}}}$ = −0.074). Example clones are displayed in different colours. *k*_p_ = 0.09 h^−1^, *k*_n_ = 0 h^-1^.

Supplementary Video 5 Confocal time-lapse imaging of the apical surface of a neural tube that mosaically expresses membrane GFP at E10.5. The first subapical *z* section (shown) was used for tracing and quantification.

Supplementary Video 6 Laser ablation experiment at E8.5 of development. Anterior, left; dorsal, up. Scale bar; 10 μm. Time reported in seconds.

Supplementary Video 7 Laser ablation experiment at E10.5 of development. Anterior, left; dorsal, up. Scale bar, 10 μm. Time reported in seconds.

Supplementary Data 1 Source data for Supplementary Figs. 1–8.

## Data Availability

Source data are available for this paper. All other data that support the plots within this paper and other findings of this study are available from the corresponding authors upon reasonable request.

## Source data

Source Data Fig. 1 Statistical source data.
Source Data Fig. 2 Statistical source data.
Source Data Fig. 3 Statistical source data.
Source Data Fig. 4 Statistical source data.
Source Data Fig. 5 Statistical source data.
Source Data Extended Data Fig. 1 Statistical source data.
Source Data Extended Data Fig. 2 Statistical source data.
Source Data Extended Data Fig. 3 Statistical source data.

## Code availability

The vertex model code used in this study is available via GitHub at https://github.com/mpzagorski/vertex_model_python_3. Other code supporting the analysis in this paper is available from the corresponding authors on reasonable request.

## References

[1] Wang X (2020). Anisotropy links cell shapes to tissue flow during convergent extension. Proc. Natl Acad. Sci. USA.

[2] Mongera A (2018). A fluid-to-solid jamming transition underlies vertebrate body axis elongation. Nature.

[3] Petridou NI, Grigolon S, Salbreux G, Hannezo E, Heisenberg CP (2019). Fluidization-mediated tissue spreading by mitotic cell rounding and non-canonical Wnt signalling. Nat. Cell Biol..

[4] Park JA (2015). Unjamming and cell shape in the asthmatic airway epithelium. Nat. Mater..

[5] Zhou J, Kim HY, Davidson LA (2009). Actomyosin stiffens the vertebrate embryo during crucial stages of elongation and neural tube closure. Development.

[6] Kim S, Pochitaloff M, Stooke-Vaughan GA, Campàs O (2021). Embryonic tissues as active foams. Nat. Phys..

[7] Bi D, Lopez JH, Schwarz JM, Manning ML (2015). A density-independent rigidity transition in biological tissues. Nat. Phys..

[8] Bi D, Yang X, Marchetti MC, Manning ML (2016). Motility-driven glass and jamming transitions in biological tissues. Phys. Rev. X.

[9] Ranft J (2010). Fluidization of tissues by cell division and apoptosis. Proc. Natl Acad. Sci. USA.

[10] Devany, J., Sussman, D. M., Yamamoto, T., Manning, M. L. & Gardel, M. L. Cell cycle–dependent active stress drives epithelia remodeling. *Proc. Natl Acad. Sci. USA* **118**, e1917853118 (2021). 10.1073/pnas.1917853118 PMC7958291 33649197

[11] Firmino J, Rocancourt D, Saadaoui M, Moreau C, Gros J (2016). Cell division drives epithelial cell rearrangements during gastrulation in chick. Dev. Cell.

[12] Matoz-Fernandez DA, Martens K, Sknepnek R, Barrat JL, Henkes S (2017). Cell division and death inhibit glassy behaviour of confluent tissues. Soft Matter.

[13] Sausedo RA, Smith JL, Schoenwolf GC (1997). Role of nonrandomly oriented cell division in shaping and bending of the neural plate. J. Comp. Neurol..

[14] Williams M, Yen W, Lu X, Sutherland A (2014). Distinct apical and basolateral mechanisms drive planar cell polarity-dependent convergent extension of the mouse neural plate. Dev. Cell.

[15] Nishimura T, Honda H, Takeichi M (2012). Planar cell polarity links axes of spatial dynamics in neural-tube closure. Cell.

[16] Zong H, Espinosa JS, Su HH, Muzumdar MD, Luo L (2005). Mosaic analysis with double markers in mice. Cell.

[17] Hippenmeyer S (2010). Genetic mosaic dissection of Lis1 and Ndel1 in neuronal migration. Neuron.

[18] Kicheva A (2014). Coordination of progenitor specification and growth in mouse and chick spinal cord. Science.

[19] Ramanathan SP, Krajnc M, Gibson MC (2019). Cell-size pleomorphism drives aberrant clone dispersal in proliferating epithelia. Dev. Cell.

[20] Dekoninck S (2020). Defining the design principles of skin epidermis postnatal growth. Cell.

[21] Guerrero P (2019). Neuronal differentiation influences progenitor arrangement in the vertebrate neuroepithelium. Development.

[22] Farhadifar R, Röper J-C, Aigouy B, Eaton S, Jülicher F (2007). The influence of cell mechanics, cell-cell interactions, and proliferation on epithelial packing. Curr. Biol..

[23] Curran S (2017). Myosin II controls junction fluctuations to guide epithelial tissue ordering. Dev. Cell.

[24] Staple DB (2010). Mechanics and remodelling of cell packings in epithelia. Eur. Phys. J..

[25] Keys AS, Abate AR, Glotzer SC, Durian DJ (2007). Measurement of growing dynamical length scales and prediction of the jamming transition in a granular material. Nat. Phys..

[26] Sussman, D. M., Paoluzzi, M., Cristina Marchetti, M. & Lisa Manning, M. Anomalous glassy dynamics in simple models of dense biological tissue. *EPL* **121**, 36001 (2018).

[27] Kursawe J, Baker RE, Fletcher AG (2018). Approximate Bayesian computation reveals the importance of repeated measurements for parameterising cell-based models of growing tissues. J. Theor. Biol..

[28] Ishihara S, Sugimura K (2012). Bayesian inference of force dynamics during morphogenesis. J. Theor. Biol..

[29] Butler MB (2019). Rho kinase-dependent apical constriction counteracts M-phase apical expansion to enable mouse neural tube closure. J. Cell Sci..

[30] Nikolopoulou E, Galea GL, Rolo A, Greene NDE, Copp AJ (2017). Neural tube closure: cellular, molecular and biomechanical mechanisms. Development.

[31] Leise WF, Mueller PR (2004). Inhibition of the cell cycle is required for convergent extension of the paraxial mesoderm during *Xenopus* neurulation. Development.

[32] Petridou NI, Heisenberg C (2019). Tissue rheology in embryonic organization. EMBO J..

[33] Barriga EH, Franze K, Charras G, Mayor R (2018). Tissue stiffening coordinates morphogenesis by triggering collective cell migration in vivo. Nature.

[34] Yan L, Bi D (2019). Multicellular rosettes drive fluid-solid transition in epithelial tissues. Phys. Rev. X.

[35] Garcia S (2015). Physics of active jamming during collective cellular motion in a monolayer. Proc. Natl Acad. Sci. USA.

[36] David R (2014). Tissue cohesion and the mechanics of cell rearrangement. Development.

[37] Erdemci-Tandogan G, Lisa Manning M (2021). Effect of cellular rearrangement time delays on the rheology of vertex models for confluent tissues. PLoS Comput. Biol..

[38] Das A, Sastry S, Bi D (2021). Controlled neighbor exchanges drive glassy behavior, intermittency, and cell streaming in epithelial tissues. Phys. Rev. X.

[39] Krajnc M, Stern T, Zankoc C (2021). Active instability and nonlinear dynamics of cell-cell junctions. Phys. Rev. Lett..

[40] Krajnc M, Dasgupta S, Ziherl P, Prost J (2018). Fluidization of epithelial sheets by active cell rearrangements. Phys. Rev. E.

[41] Czajkowski M, Sussman DM, Marchetti MC, Manning ML (2019). Glassy dynamics in models of confluent tissue with mitosis and apoptosis. Soft Matter.

[42] Nematbakhsh A (2017). Multi-scale computational study of the mechanical regulation of cell mitotic rounding in epithelia. PLoS Comput. Biol..

[43] Malmi-Kakkada AN, Li X, Samanta HS, Sinha S, Thirumalai D (2018). Cell growth rate dictates the onset of glass to fluidlike transition and long time superdiffusion in an evolving cell colony. Phys. Rev. X.

[44] Lange C, Calegari F (2010). Cdks and cyclins link G_1_ length and differentiation of embryonic, neural and hematopoietic stem cells. Cell Cycle.

[45] Wartlick O (2011). Dynamics of Dpp signaling and proliferation control. Science.

[46] Marcon, L., Arqués, C. G., Torres, M. S. & Sharpe, J. A computational clonal analysis of the developing mouse limb bud. *PLoS Comput. Biol*. **7**, e1001071 (2011). 10.1371/journal.pcbi.1001071 PMC3037386 21347315

[47] Strzyz PJ, Matejcic M, Norden C (2016). Heterogeneity, cell biology and tissue mechanics of pseudostratified epithelia: coordination of cell divisions and growth in tightly packed tissues. Int. Rev. Cell Mol. Biol..

[48] Cammarota CM, Bergstralh D (2020). Cell division: interkinetic nuclear… mechanics. Curr. Biol..

[49] Kirkland NJ (2020). Tissue mechanics regulate mitotic nuclear dynamics during epithelial development. Curr. Biol..

[50] Livet J (2007). Transgenic strategies for combinatorial expression of fluorescent proteins in the nervous system. Nature.

[51] Arnold K (2011). Sox2+ adult stem/progenitor cells are important for tissue regeneration and survival of mice. Cell Stem Cell.

[52] Muzumdar MD, Tasic B, Miyamichi K, Li L, Luo L (2007). A global double-fluorescent Cre reporter mouse. Genesis.

[53] Katsunuma S (2016). Synergistic action of nectins and cadherins generates the mosaic cellular pattern of the olfactory epithelium. J. Cell Biol..

[54] Madisen L (2010). A robust and high-throughput Cre reporting and characterization system for the whole mouse brain. Nat. Neurosci..

[55] Schindelin J (2012). Fiji: an open-source platform for biological-image analysis. Nat. Methods.

[56] Balaskas N (2012). Gene regulatory logic for reading the Sonic Hedgehog signaling gradient in the vertebrate neural tube. Cell.

[57] Udan RS, Piazza VG, Hsu C, Hadjantonakis A, Dickinson ME (2014). Quantitative imaging of cell dynamics in mouse embryos using light-sheet microscopy. Development.

[58] Aigouy B (2010). Cell flow reorients the axis of planar polarity in the wing epithelium of *Drosophila*. Cell.

[59] Kursawe J, Baker RE, Fletcher AG (2017). Impact of implementation choices on quantitative predictions of cell-based computational models. J. Comput. Phys..

