# Supplementary material for: Cell cycle dynamics control fluidity of the developing mouse neuroepithelium
Source: Nat Phys. 2023 Apr 6;19(7):1050–8. doi: 10.1038/s41567-023-01977-w (PMC10344780; doi:10.1038/s41567-023-01977-w)
Supplement: Supplementary file 1 — Supplementary Figs. 1–8 and Tables 1–3. [file 41567_2023_1977_MOESM1_ESM.pdf]

nature physics

Article

https://doi.org/10.1038/s41567-023-01977-w

Cell cycle dynamics control fluidity of the developing mouse neuroepithelium

In the format provided by the authors and unedited

Table of Contents

Supplementary figures.........2

Figure S1. MADM clonal labelling in the mouse spinal cord.........2

Figure S2. Cell shape descriptors in simulations.........3

Figure S3. Relationships between T1 transitions and cell divisions in simulations.........4

Figure S4. Relationship between cell divisions and cell rearrangements in the neural tube.........5

Figure S5. Cell shapes at different stages of neural tube development.........6

Figure S6. Fragmentation coefficient, T1 rate and cell area CV for different proliferation rates.........7

Figure S7. Cell proliferation and differentiation affect clone fragmentation.........8

Figure S8. The proliferation rate affects tissue morphogenesis.........9

Supplementary tables.........10

Table S1. Sample sizes.........10

Table S2. Vertex model parameters in simulations.........12

Table S3. Cell shape descriptors and their definition.........13

Supplementary figures

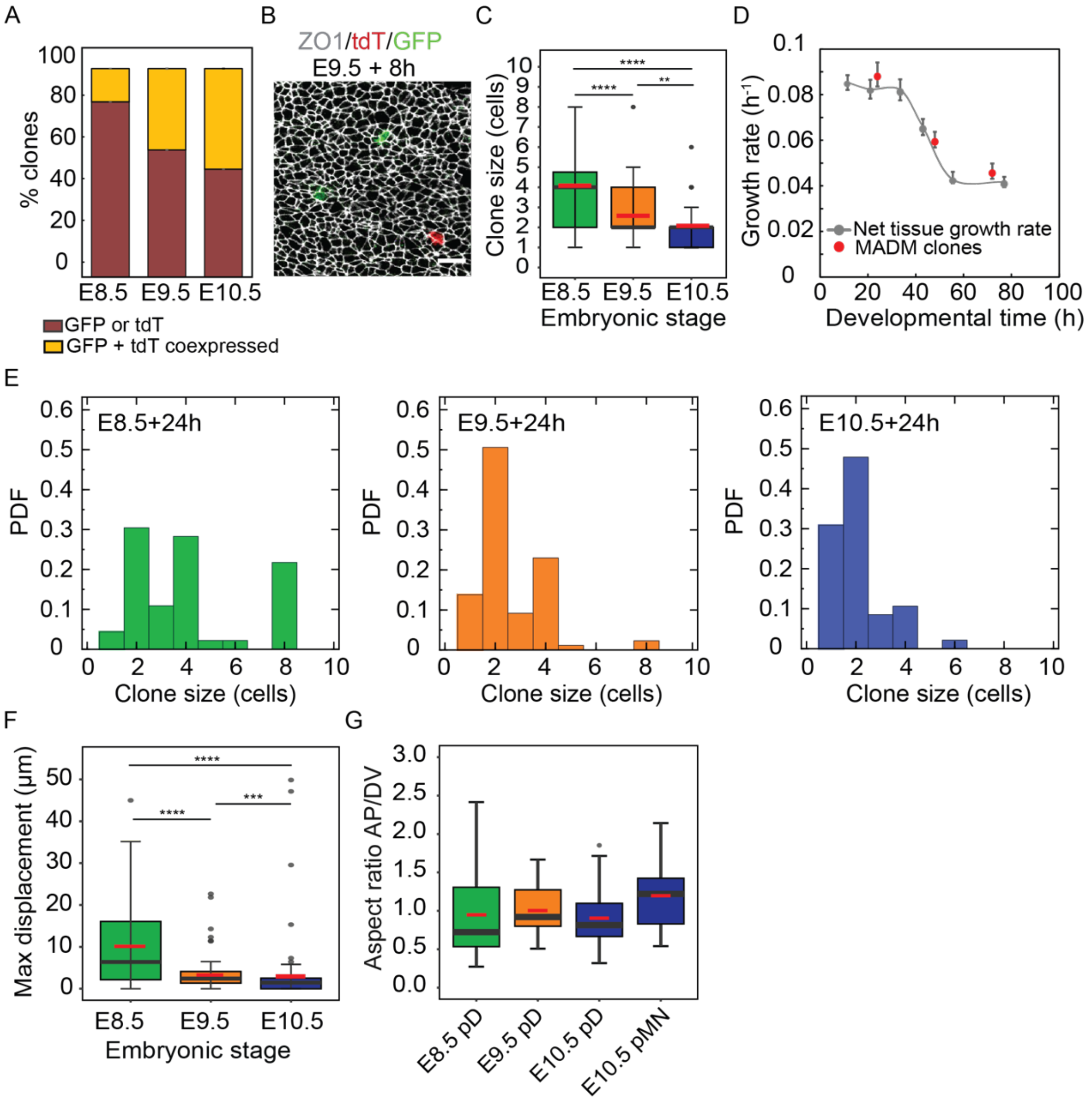

Figure S1. MADM clonal labelling in the mouse spinal cord. **A.** Fraction of MADM clones resulting from G2-X segregation (brown, daughter cells express either GFP or tdTomato), or G2-Z or G1 recombination (yellow, daughter cells co-express GFP and tdTomato). **B.** Confetti clones after 8h of tamofixen induction at E9.5. Immunostaining against RFP, EGFP and ZO1. Scale bar, 10 µm. **C.** Mean sizes of MADM clones induced at the indicated stages and analyzed 24h later. 25-75th percentile (box), median (black), mean (red), highest/lowest observations without outliers (whiskers). Mann-Whitney test (two-sided): P values = 0.0001 (E8.5 vs E9.5), 9.4e-09 (E8.5 vs E10.5), 0.0024 (E9.5 vs E10.5). Sample sizes in C-F (number of clones): E8.5 n = 46, E9.5 n = 87, E10.5 n = 94 (see also Table S1). **D.** Growth rate measured from the net growth rate of the tissue in [18] (grey) and predicted from the mean MADM clone size (red, Methods). Error bars, mean $\pm$ SEM. **E.** Clone size distributions at different stages. **F.** Maximum displacement of labelled cells from the clone centroid for clones induced at E8.5, E9.5 and E10.5 (values correspond to 3.1±0.4, 1.1±0.1 and 1.2±0.3 cell diameters, respectively). Mann-Whitney test (two-sided) P = 3.3e-05 (E8.5 vs E9.5), 8.4e-09 (E8.5 vs E10.5), 0.0006 (E9.5 vs E10.5). Box-and-whisker plots as in C. **G.** AP/DV aspect ratio of clones at different stages and domains. Clones in the p3 domain and floor plate were excluded from analysis. Mann-Whitney test (two-sided) $P > 0.05$ for all pairwise comparisons. Sample sizes: n= 31, 57, 56, 6 clones (left to right; Table S1). Box plots as in C.

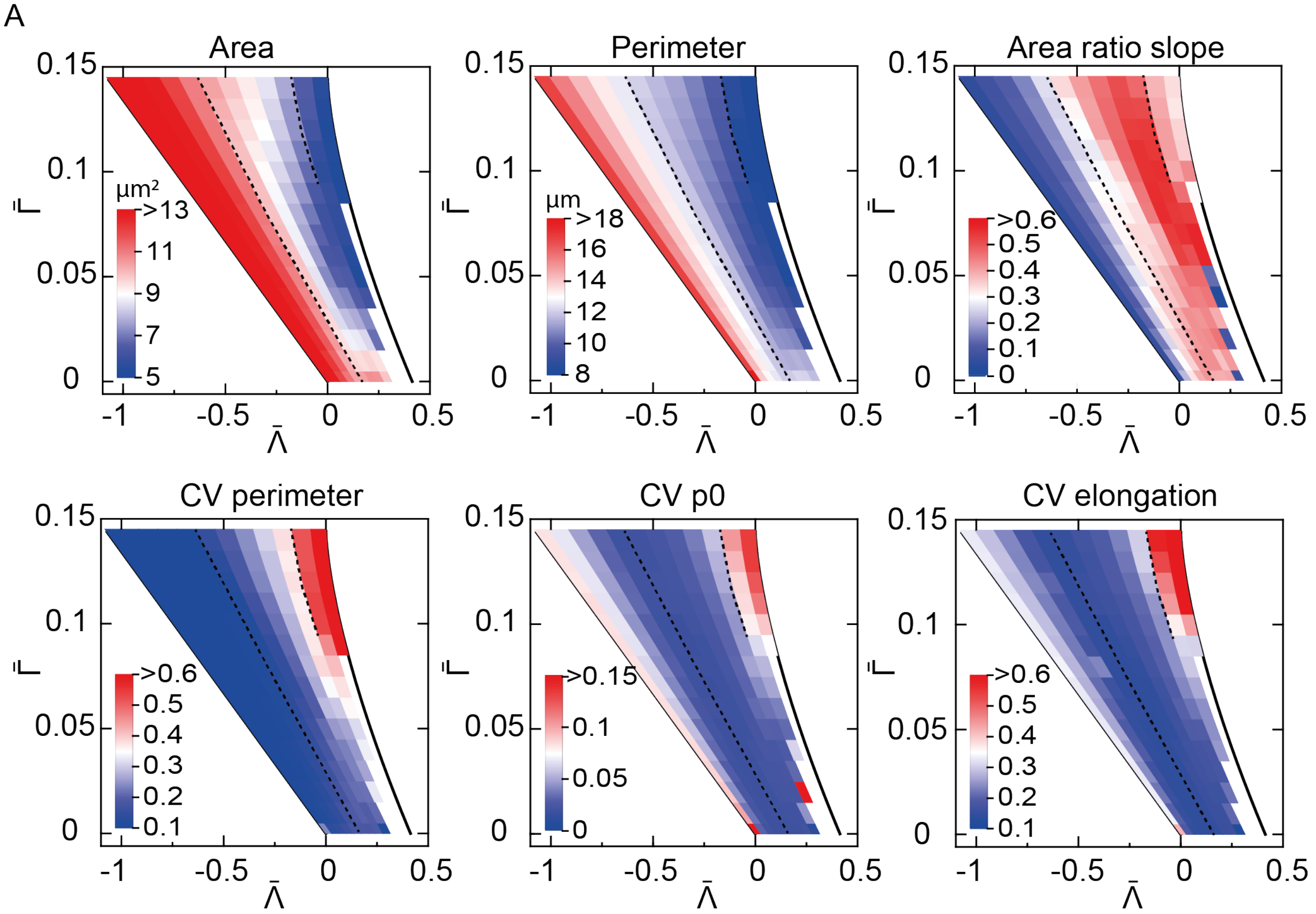

Figure S2. Cell shape descriptors in simulations. **A.** Mean value of the indicated cell shape descriptors (defined in Table S3) for simulations performed at different values of $\bar{\Lambda}$ and $\bar{\Gamma}$ for proliferation rate k_p = 0.09h^{-1} and differentiation rate k_n = 0. The mean value was color-coded with scale indicated in insets of respective panels. The mean was estimated from all cells pooled together from 10 simulations for each pair of ($\bar{\Lambda}$, $\bar{\Gamma}$) at the final time.

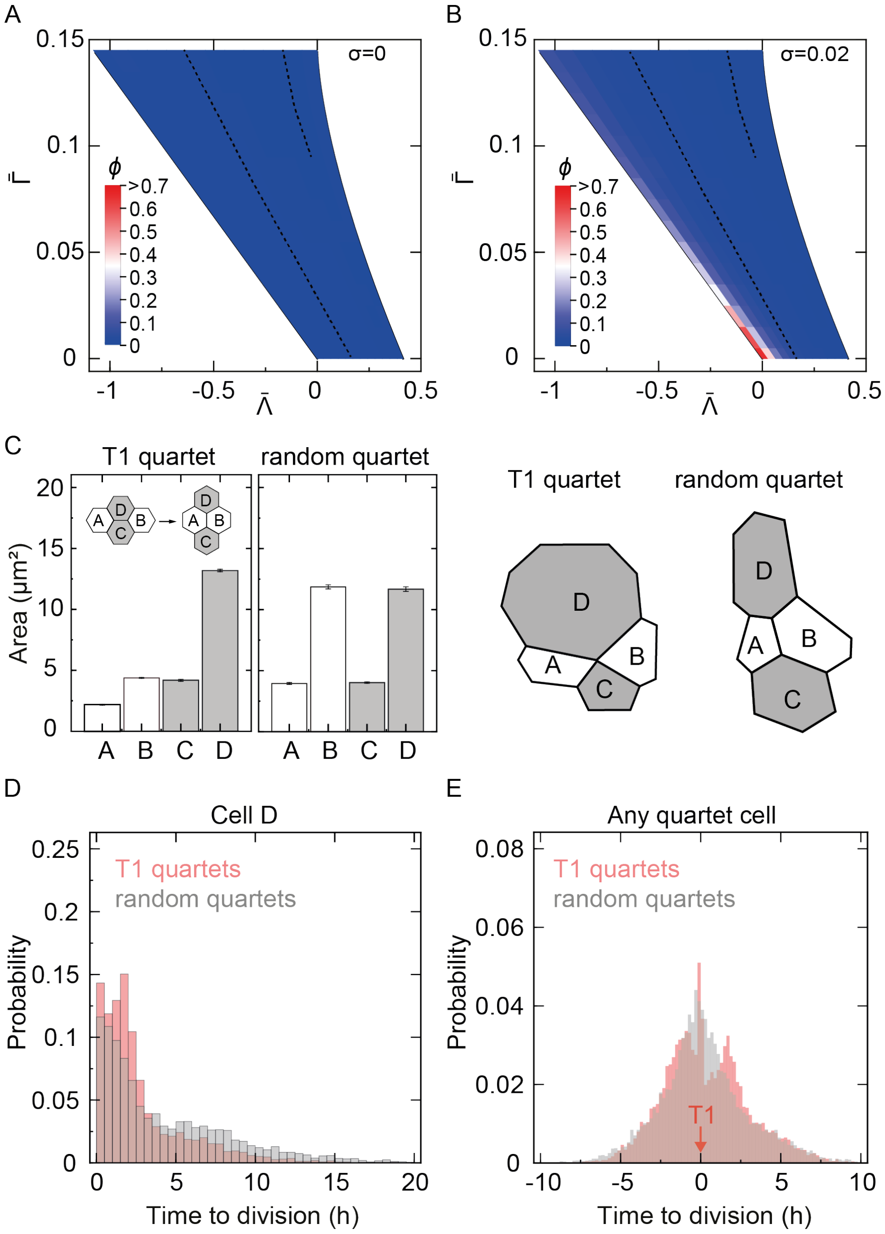

Figure S3. Relationships between T1 transitions and cell divisions in simulations. **A-B.** Simulations without IKNM effect (linear area increase, k_p = 0.09h^{-1}) result in non-fragmented clones. Coefficient of fragmentation for model without line tension noise (σ = 0) (A) and with σ = 0.02 (B). Dashed lines indicate the boundaries of regions A, B and C as in Fig. 3A. **C.** Mean apical cell area of cells in T1 or randomly selected quartets in simulations of region *C*. Cells in the quartets are designated A, B, C, D as indicated in the schematic and are further ordered by size (apical area), so that A<B, C<D. On the right are examples of T1 and random quartets. Error bars, SEM from 10 independent simulations with >6000 quartets each. For all pairwise comparisons between T1 and random quartets, two-sided *t*-test $P < 0.05$. **D.** Distribution of time intervals from the T1 event (or a random time point in random quartets) to cell division of the largest cell, cell D. **E.** Distribution of time intervals between T1 event (or random time point) and cell division of any cell in the quartet. Simulation parameters $\overline{\Gamma}$ = 0.12 and $\overline{\Lambda}$ = -0.074 (Region C). The T1 events (n=7387) and random quartets (n=5986) were considered in a 16h time window with 8h buffer intervals to avoid boundary effects.

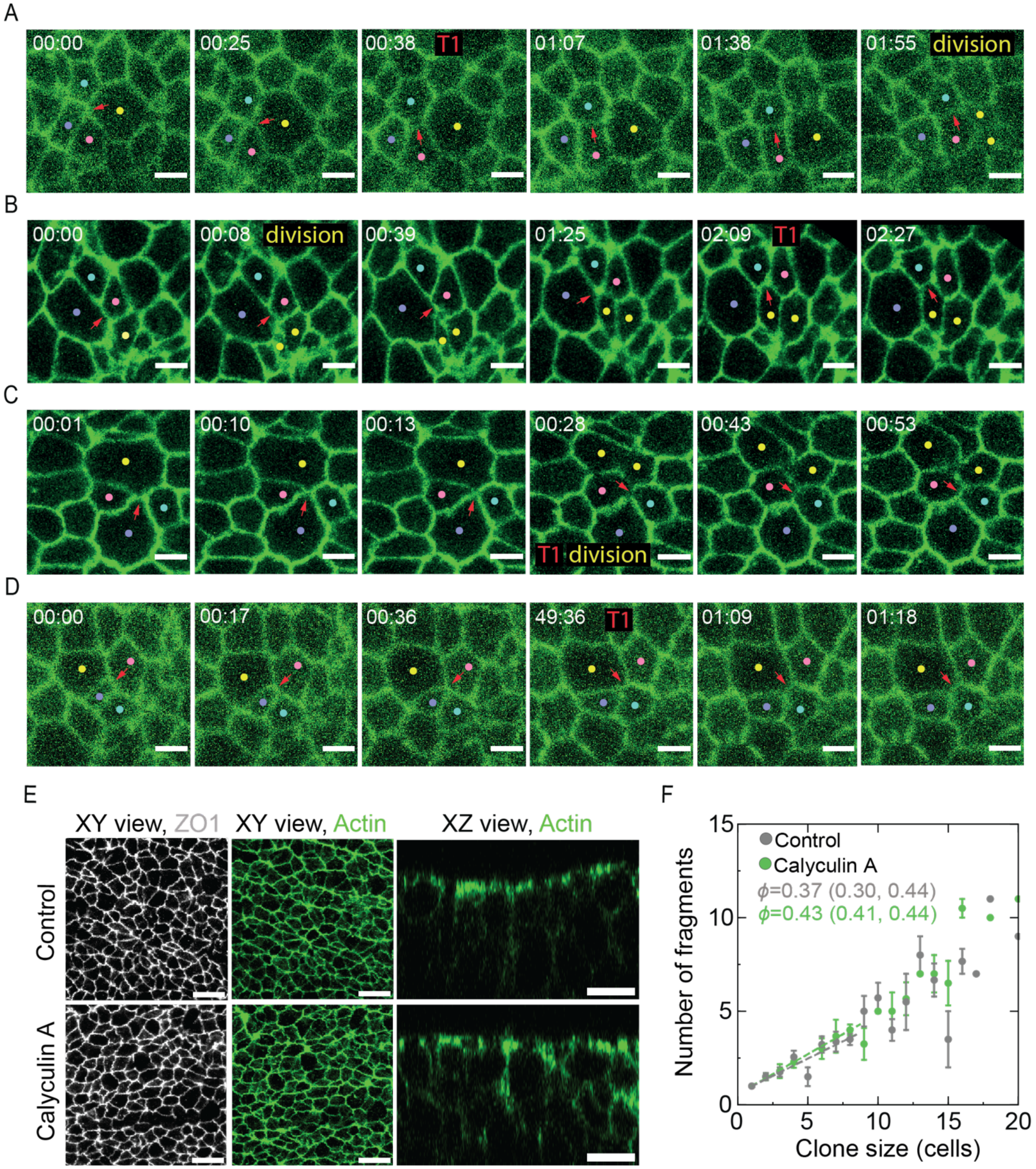

Figure S4. Relationship between cell divisions and cell rearrangements in the neural tube. **A-D.** Examples of time-lapse imaging of ZO1-GFP (green) expressing neural plates at E8.5. Time is shown as hh:mm. The time points of T1 transitions and cell divisions (cytokinesis) are indicated. Cell participating in the T1 transition are marked with dots, the T1 junction that changes orientation is indicated with a red arrow. The dividing cell is marked in yellow. In D, the yellow cell increases in area but does not divide in the time interval of the video. Scale bars, 5 µm. **E.** Apical view of neural epithelium treated with 0.6nM of Calyculin A or vehicle from E8.5 for 42 hours. After culture the tissue was stained for F-Actin and ZO1. Increase in the level of actin was observed along the basolateral sides of Calyculin A treated cells. Scale bars, 10 µm. **F.** The fragmentation of Confetti clones upon Calyculin A treatment is similar to control. The fragmentation coefficient ϕ (95% CI) was obtained using linear fit to the data for clones ≤8 cells (dashed lines). Sample sizes: Control n= 87, Cal A n = 69 clones. Error bars, SEM.

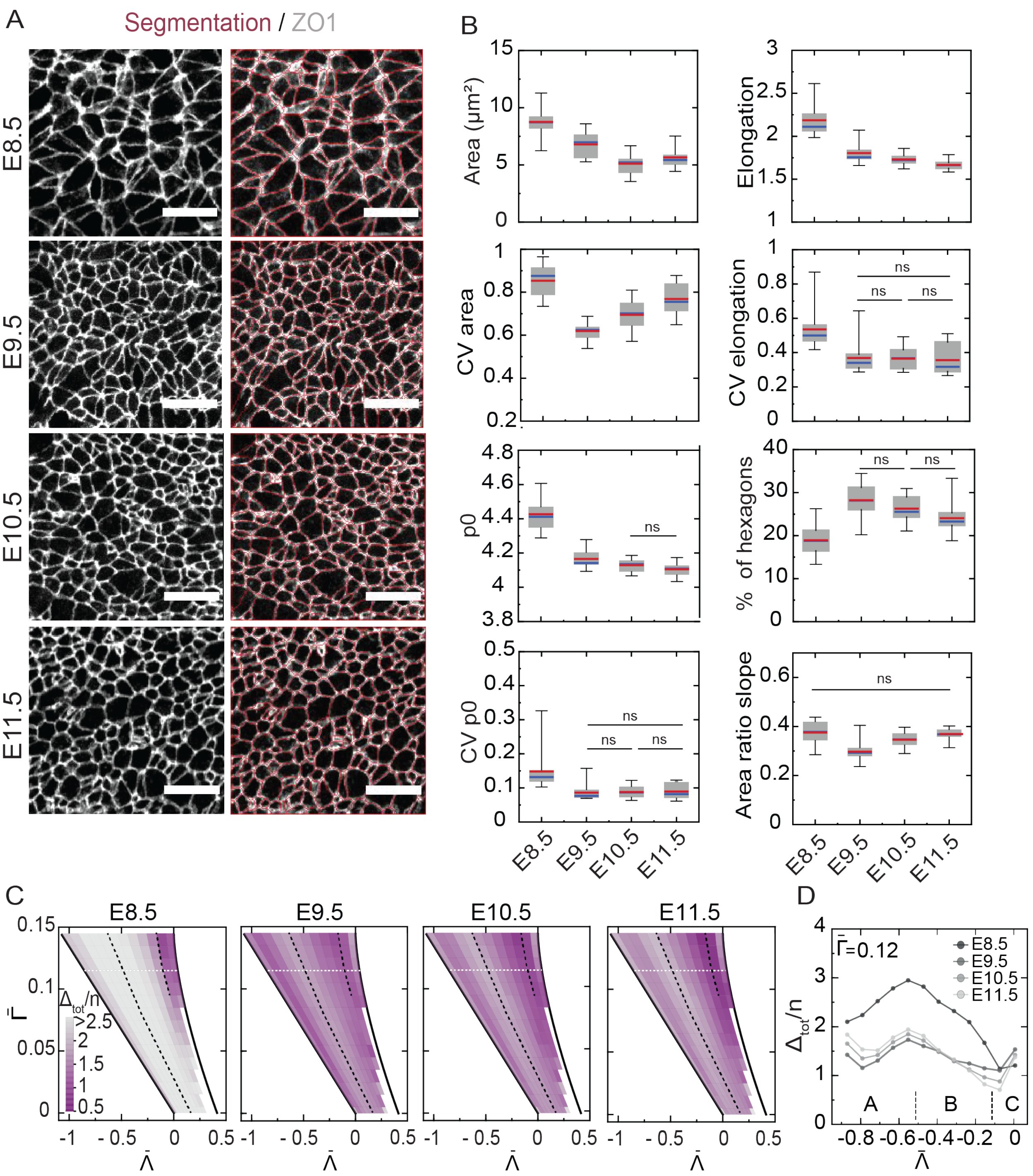

Figure S5. Cell shapes at different stages of neural tube development. **A**. Top, apical view of the epithelium with ZO1 immunostaining (white). Bottom, cell segmentation (red traces). Scale bars, 10 µm. **B**. Quantification of the indicated cell shape descriptors at different stages. 25-75th percentile (box), median (blue), mean (red), highest/lowest observations (whiskers). For all pairwise comparisons, two sided *t*-tests were $P < 0.05$, except for the cases that are shown as ns ($P > 0.05$). Samples sizes (number of cells): E8.5 n= 6929, E9.5 n= 2433, E10.5 n= 5520, E11.5 n= 3583 (see also Table S1). **C-D.** Difference between the cumulative distribution of cell shape descriptors p_0, ϵ, α, hex, $p_{0_{CV}}$, ε_{CV}, A_{CV}, and P_{CV} (defined in Table S3) in simulations and experimental data (color-coded). In D, the values are shown numerically for $\bar{\Gamma} = 0.12$ (white dashed line in C). Black dashed lines – borders between regions A, B, C as defined in Fig. 3A.

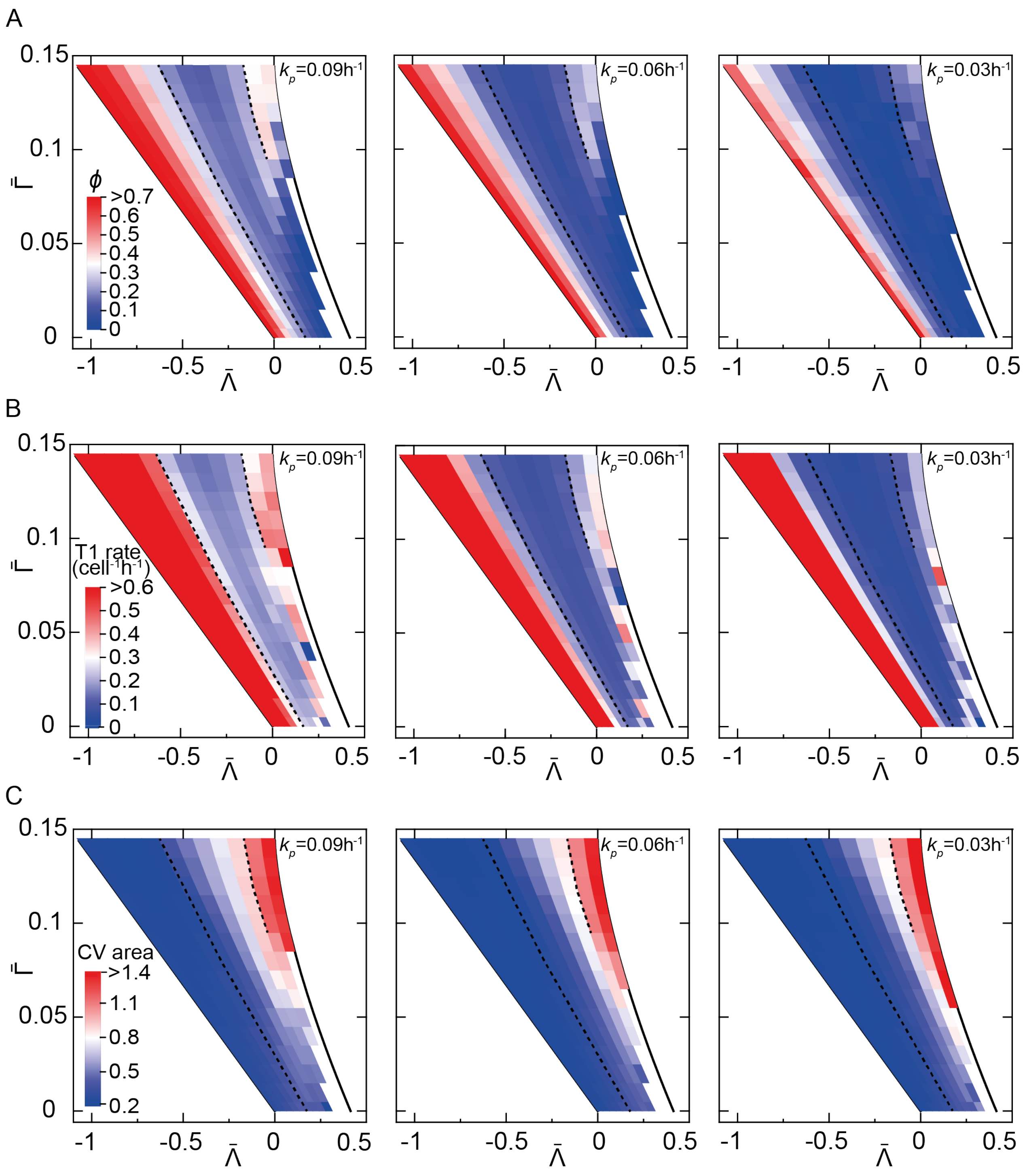

Figure S6. Fragmentation coefficient, T1 rate and cell area CV for different proliferation rates. **A.** Fragmentation coefficient at different values of $\overline{\Lambda}$, $\overline{\Gamma}$ for high (k_p = 0.09h^{-1}), intermediate (k_p = 0.06h^{-1}) and low (k_p = 0.03h^{-1}) rates of proliferation. Differentiation rate k_n = 0. **B.** Rate of T1 transitions with the same set of parameters as in A. **C.** The coefficient of variation of cell areas with the same set of parameters as in A. The estimates are mean values from 10 simulations for each pair of ($\overline{\Lambda}$, $\overline{\Gamma}$) at the final time.

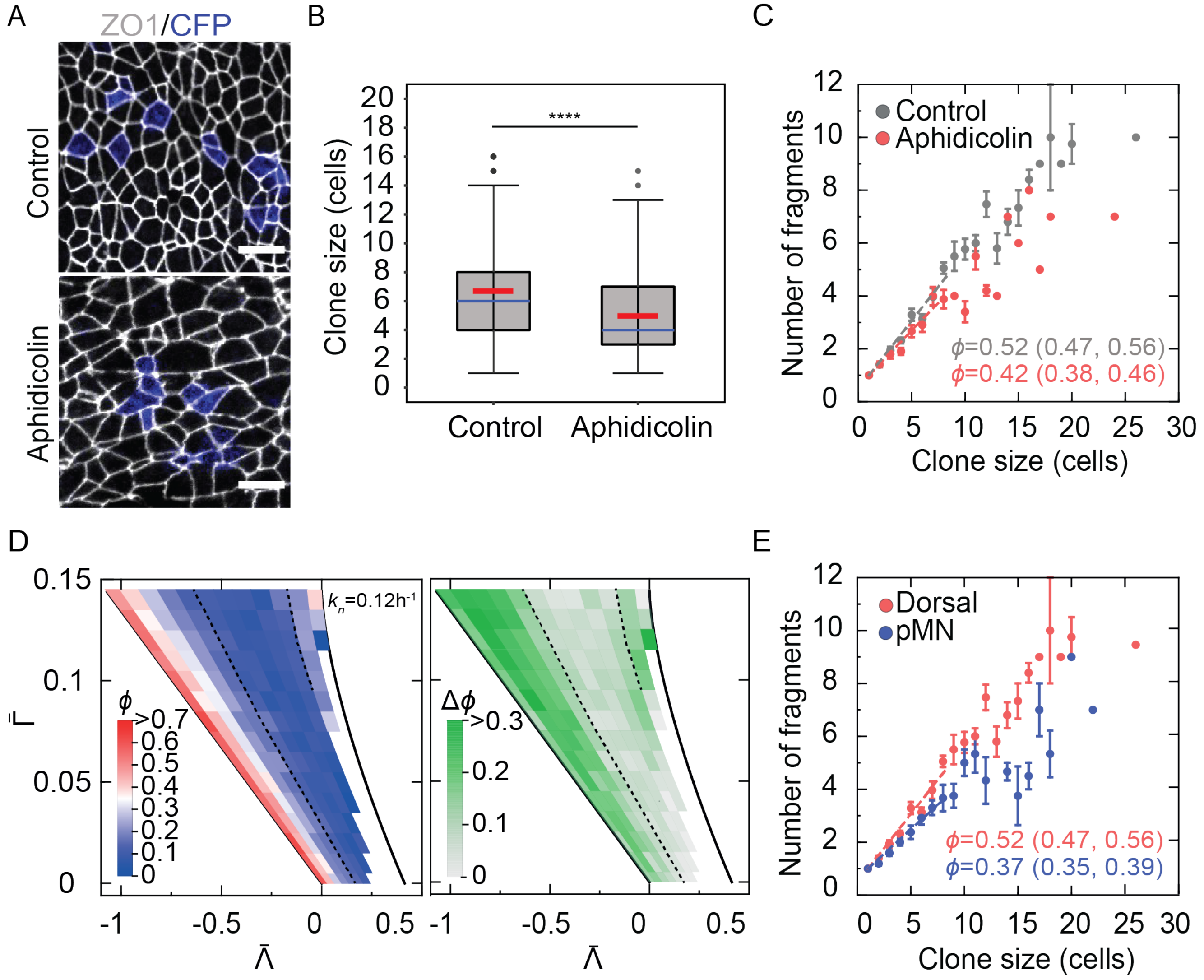

Figure S7. Cell proliferation and differentiation affect clone fragmentation**. A.** Confetti clones (CFP) from embryos injected with tamoxifen at E7.5 and cultured *ex utero* from E8.5 for 42h with 800nM aphidicolin or control medium. Scale bars, 10 µm. **B.** Clone size for the conditions in A. 25-75th percentile (box), median (blue), mean (red), highest/lowest observations without outliers (whiskers). Mann-Whitney test (two-sided), P = 3e-05. Samples sizes (B,C): Control n = 382 clones, Aphidicolin n = 185 clones (see also Table S1). **C.** Mean number of fragments per clone for a given clone size for control vs aphidicolin treated embryos. The fragmentation coefficient ϕ (95% CI) was obtained using linear fit to the data for clones ≤8 cells (dashed lines). Error bars, SEM. **D**. ϕ for simulations with high differentiation rate k_n = 0.12h^{-1} and k_p = 0.09h^{-1} (left). Right, difference in ϕ between k_n = 0 (Fig. 3A) and k_n = 0.12h^{-1}. **E.** Mean number of fragments per clone $\pm$ SEM for Confetti clones in the dorsal (n = 319 clones) vs pMN (n = 190 clones) domain of embryos from the control condition in Fig. 5E-G. The fragmentation coefficient ϕ (95% CI) was obtained using linear fit to the data for clones ≤8 cells (dashed lines).

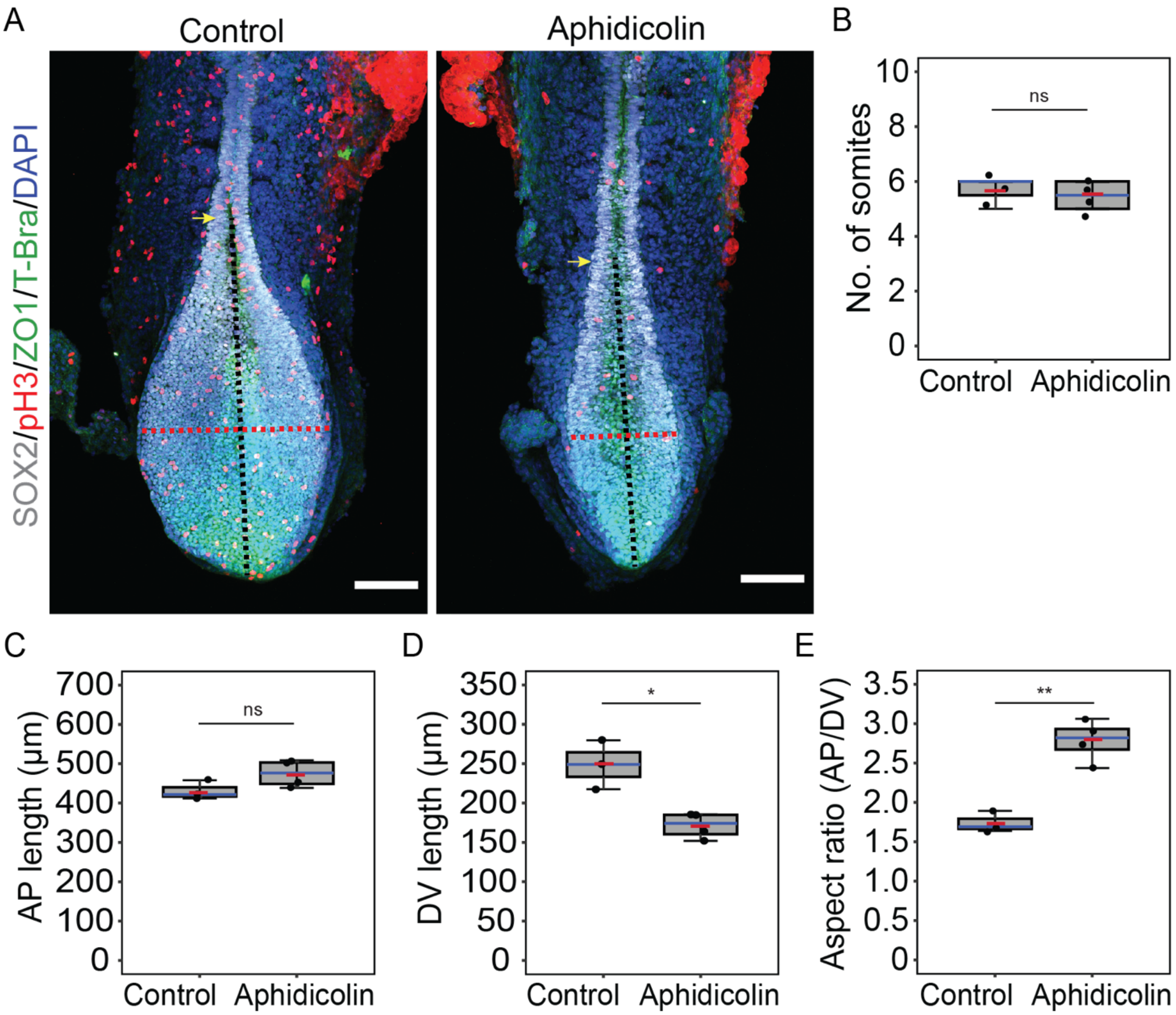

Figure S8. The proliferation rate affects tissue morphogenesis. **A.** Neural plate and trunk region of E8.5 embryos treated with vehicle or 5µM aphidicolin for 8 hours. Yellow arrow indicates the border of the last formed pair of somites. Scale bar, 100 µm. Anterior, up; dorsal view. **B.** Number of somites in the analysed embryos. (B-E) 25-75th percentile (box), median (blue), mean (red), highest/lowest observations without outliers (whiskers). Two sided *t*-test ns, $P = 0.72$. **C.** Anterior-posterior length of the neural plate, measured along the black dashed line in A (from the somite border to the caudal tip). Two sided *t*-test ns, $P = 0.1$. **D.** Dorso-ventral length of the neural plate measured along the red line in A (maximum width). Two sided *t*-test $P = 0.033$. **E.** Aspect ratio of the neural plate (AP/DV lengths). Two sided *t*-test $P = 0.0014$. Sample size (B-E): Control n = 3 embryos, Aphidicolin n = 4 embryos.

Supplementary tables

Table S1. Sample sizes.

Number of features and number of embryos these features were quantified from.

A. Number of MADM clones used to quantify ϕ at the indicated stages (relevant to Fig. 1 and Fig. S1A-F).

Condition	Number of clones	Number of embryos	Number of litters
MADM E8.5 + 24h	46	27	10
MADM E9.5 + 24h	87	19	4
MADM E10.5 + 24h	94	32	6

B. Quantification at distinct cell cycle phases at E8.5 and E10.5 (relevant to Fig. 2A-C).

Stage	Cell cycle phase	Number of cells	Number of embryos
E8.5	S	172	5
E8.5	M	179	6
E10.5	S	197	6
E10.5	G2	147	5
E10.5	M	144	4

C. Edge length fluctuations in time-lapse images of ZO1-GFP embryos (relevant to Fig. 2D).

Stage	Number of edges	Number of embryos
E8.5	309	3
E10.5	387	3

D. Quantification of cell shape descriptors in Fig. 3G-H and S5.

Stage	Number of segmented cells	Number of images	Number of embryos
E8.5	6929	16	4
E9.5	2433	11	4
E10.5	5520	21	10
E11.5	3583	13	7

E. Laser ablation of cell bonds performed at E8.5 and E10.5 (relevant to Fig. 5A-C).

Stage	Number of ablated junctions	Number of embryos
E8.5	14	8
E10.5	11	7

F. Confetti clones analyzed upon inhibition of cell proliferation (relevant to Fig. 5E-G and Fig. S7A-C).

Condition	Number of clones	Number of embryos
L-mimosine	155	7
Control	382	12

Aphidicolin	185	12
Control	382	12

G. Aspect ratio of MADM clones at different developmental stages (relevant to Fig. 1SG)

Stage	Number of clones	Number of embryos
E8.5 pD	31	18
E9.5 pD	57	17
E10.5 pD	56	30
E10.5 pMN	6	6

H. Number of Confetti clones analyzed upon Calyculin A treatment (relevant to Fig. S4E-F).

Condition	Number of clones	Number of embryos
Calyculin A	69	7
Control	87	6

I. Number of Confetti clones analyzed in the pD and pMN domain of the neural tube (relevant to Fig. S7E).

Domain	Number of clones	Number of embryos
pD	319	7
pMN	190	7

J. Neural plate dimensions upon inhibition of cell proliferation (relevant to Fig. S8).

Condition	Number of embryos
Aphidicolin	3
Control	4

Table S2. Vertex model parameters in simulations

Parameter	Description	Value
Δt	Simulation time step	0.29 s
Δl	Simulation length unit	4.6 μm
K	Elasticity	1 au
$\bar{\Lambda}$	Normalized line tension	$\bar{\Gamma}$= 0, 0.01, …, 0.14<br>for each $\bar{\Gamma}$ there are 10 equally spaced $\bar{\Lambda}$ that are delimited by:<br>$-4\sqrt{2}3^{1/4}\bar{\Gamma} < \bar{\Lambda} < \frac{2\sqrt{6}}{27}(\sqrt{3} - 12\,\bar{\Gamma})^{3/2}$
$\bar{\Gamma}$	Normalized contractility	
A_c	Critical cell area	27 μm^2
l_{T1}	T1 transition length threshold	0.046 μm
l_{new}	Edge length after T1 transition	$1.01 l_{T1}$
μ	Medium viscosity	0.12 K μm^2 s
μ''/μ'	AP to DV drag viscosity ratio	50
t_T	total cell cycle time	8 h, 12 h, 20 h
k_p	proliferation rate, $\ln 2/t_T$	0.09 h^{-1}, 0.06 h^{-1}, 0.03 h^{-1}
k_n	terminal neuronal differentiation rate	0, 0.12 h^{-1}
σ	magnitude of noise in internal line tension	0, 0.02
τ	internal line tension correlation time	37 s
σ_g	standard deviation of cell growth rate (see eq. 3)	0.45 $/t_T$

Table S3. Cell shape descriptors and their definition

Descriptor symbol	Name	Definition
A	Cell area	Apical surface area of the cells at the level of tight junctions (μm^2)
P	Cell perimeter	Perimeter of the cells at the level of tight junctions (μm)
p_0	Cell shape index	$p_0 = P/\sqrt{A}$
ε	Cell elongation	$\sqrt{v1/v2}$, where $v1$ and $v2$ are the eigenvalues of the second moment matrix of the vertices of the cell, $v1 \geq v2$
A_{CV}	Cell area CV	Coefficient of variation of *A*
P_{CV}	Cell perimeter CV	Coefficient of variation of *P*
p_{0_CV}	Cell shape index CV	Coefficient of variation of p_0
ε_{CV}	Cell elongation CV	Coefficient of variation of ε
hex	Fraction of hexagons	Ratio of hexagons to all polygons in the tissue
α	Area ratio slope	Determined from a fit $\frac{\langle A_n \rangle}{\langle A \rangle} = \alpha(n-3) + b$, where $\langle A_n \rangle$ is the mean area of n-sided cells and n is the number of sides, $4 \leq n \leq 8$
