# Supplementary material for: Cell cycle dynamics control fluidity of the developing mouse neuroepithelium
Source: Nat Phys. 2023 Apr 6;19(7):1050–8. doi: 10.1038/s41567-023-01977-w (PMC10344780; doi:10.1038/s41567-023-01977-w)
Supplement: Supplementary file 2 — Reporting Summary [file 41567_2023_1977_MOESM2_ESM.pdf]

nature portfolio

Corresponding author(s): Marcin Zagorski and Anna Kicheva
Last updated by author(s): Jan 24, 2023

Reporting Summary

Nature Portfolio wishes to improve the reproducibility of the work that we publish. This form provides structure for consistency and transparency in reporting. For further information on Nature Portfolio policies, see our Editorial Policies and the Editorial Policy Checklist.

Statistics

For all statistical analyses, confirm that the following items are present in the figure legend, table legend, main text, or Methods section.

n/a	Confirmed	
☐	☒	The exact sample size (n) for each experimental group/condition, given as a discrete number and unit of measurement
☐	☒	A statement on whether measurements were taken from distinct samples or whether the same sample was measured repeatedly
☐	☒	The statistical test(s) used AND whether they are one- or two-sided<br>*Only common tests should be described solely by name; describe more complex techniques in the Methods section.*
☐	☒	A description of all covariates tested
☐	☒	A description of any assumptions or corrections, such as tests of normality and adjustment for multiple comparisons
☐	☒	A full description of the statistical parameters including central tendency (e.g. means) or other basic estimates (e.g. regression coefficient) AND variation (e.g. standard deviation) or associated estimates of uncertainty (e.g. confidence intervals)
☐	☒	For null hypothesis testing, the test statistic (e.g. F, t, r) with confidence intervals, effect sizes, degrees of freedom and P value noted<br>*Give P values as exact values whenever suitable.*
☒	☐	For Bayesian analysis, information on the choice of priors and Markov chain Monte Carlo settings
☒	☐	For hierarchical and complex designs, identification of the appropriate level for tests and full reporting of outcomes
☒	☐	Estimates of effect sizes (e.g. Cohen's d, Pearson's r), indicating how they were calculated

Our web collection on statistics for biologists contains articles on many of the points above.

Software and code

Policy information about availability of computer code

Data collection	LSM880 inverted with Zen Black 2.3 software was used to collect clone images.<br>LSM800 inverted with Zen Blue 3.5 software was used for live imaging.<br>Andor spinning disc system with inverted Axio Observer Z1 was used for junction ablations.
Data analysis	Clone images and junction ablations movies were analyzed with Fiji.<br>Live imaging files were analyzed with Fiji and Imaris 9.1.<br>Cell segmentation was performed using Tissue Analyzer plugin in Fiji.<br>Analysis of vertex model results was performed using Mathematica 12.1. The code used for vertex model simulations is available in GitHub

For manuscripts utilizing custom algorithms or software that are central to the research but not yet described in published literature, software must be made available to editors and reviewers. We strongly encourage code deposition in a community repository (e.g. GitHub). See the Nature Portfolio guidelines for submitting code & software for further information.

Data

Policy information about availability of data

All manuscripts must include a data availability statement. This statement should provide the following information, where applicable:

- Accession codes, unique identifiers, or web links for publicly available datasets
- A description of any restrictions on data availability
- For clinical datasets or third party data, please ensure that the statement adheres to our policy

The datasets that support the current study are available from the corresponding authors on reasonable request (as stated in the Data Availability Statement).

Human research participants

Policy information about studies involving human research participants and Sex and Gender in Research.

Reporting on sex and gender	*Use the terms sex (biological attribute) and gender (shaped by social and cultural circumstances) carefully in order to avoid confusing both terms. Indicate if findings apply to only one sex or gender; describe whether sex and gender were considered in study design whether sex and/or gender was determined based on self-reporting or assigned and methods used. Provide in the source data disaggregated sex and gender data where this information has been collected, and consent has been obtained for sharing of individual-level data; provide overall numbers in this Reporting Summary. Please state if this information has not been collected. Report sex- and gender-based analyses where performed, justify reasons for lack of sex- and gender-based analysis.*
Population characteristics	*Describe the covariate-relevant population characteristics of the human research participants (e.g. age, genotypic information, past and current diagnosis and treatment categories). If you filled out the behavioural & social sciences study design questions and have nothing to add here, write "See above."*
Recruitment	*Describe how participants were recruited. Outline any potential self-selection bias or other biases that may be present and how these are likely to impact results.*
Ethics oversight	*Identify the organization(s) that approved the study protocol.*

Note that full information on the approval of the study protocol must also be provided in the manuscript.

Field-specific reporting

Please select the one below that is the best fit for your research. If you are not sure, read the appropriate sections before making your selection.

☒ Life sciences ☐ Behavioural & social sciences ☐ Ecological, evolutionary & environmental sciences

For a reference copy of the document with all sections, see nature.com/documents/nr-reporting-summary-flat.pdf

Life sciences study design

All studies must disclose on these points even when the disclosure is negative.

Sample size	All sample sizes are reported in the figure legends and in the Supplementary Table S1.<br>Data was collected from experiments that involve breeding of transgenic mice, therefore sample sizes were minimized whenever possible. Experiments were reproduced at least three times and all transgenic embryos resulting from the experimental timed matings were taken for analysis.
Data exclusions	No data were excluded from analysis. The fragmentation coefficient analysis is constrained to clones with small sizes (as described in Methods) for which reliable statistics can be obtained.
Replication	We confirm that all experimental results have been repeated reproducibly at least 3 times. Simulations were performed 10 times per condition, as described in the Methods and figure legends.
Randomization	No randomization methods were used to determine how samples/organisms were allocated.
Blinding	Blinding was not performed. The experimental data was collected and analyzed by the same person.

Reporting for specific materials, systems and methods

We require information from authors about some types of materials, experimental systems and methods used in many studies. Here, indicate whether each material, system or method listed is relevant to your study. If you are not sure if a list item applies to your research, read the appropriate section before selecting a response.

Materials & experimental systems

n/a	Involved in the study	
☐	☒	Antibodies
☒	☐	Eukaryotic cell lines
☒	☐	Palaeontology and archaeology
☐	☒	Animals and other organisms
☒	☐	Clinical data
☒	☐	Dual use research of concern

Methods

n/a	Involved in the study	
☒	☐	ChIP-seq
☒	☐	Flow cytometry
☒	☐	MRI-based neuroimaging

Antibodies

Antibodies used

Primary antibodies:
Mouse anti-ZO1 (33-9100 Invitrogen, 1:90)
Goat anti-Olig2 (AF2418 R&D systems, 1:100)
Sheep anti-GFP (4745-1051 AbD Serotec, 1:1000)
Rabbit anti-RFP (600-401-379 Rockland, 1:2000)
Mouse anti-Nkx2.2 (74.5A5 DSHB, 1:20)
Rat anti-pH3 (H9908 Sigma,1:1000)
Goat anti-SOX2 (AF2018 R&D systems, 1:100)
Rabbit anti-Brachyury (ab209665 Abcam, 1:100).

Secondary antibodies:
Donkey anti-mouse Alexa fluor 647 (Jackson Immuno, 1:250)
Donkey anti-goat FITC (Jackson Immuno, 1:250)
Donkey anti-rabbit Cy3 (Jackson Immuno, 1:1000)
Donkey anti-rat Cy3 (Jackson Immuno, 1:1000)
Donkey anti-sheep FITC (Jackson Immuno, 1:250).

Validation

All antibodies were previously validated by vendors and published work. Relevant studies include:
Mouse anti-ZO1: The tumor suppressor PTEN and the PDK1 kinase regulate formation of the columnar neural epithelium (Grego-Bessa et al. 2015 eLife DOI: 10.7554/eLife.12034)
Goat anti-Olig2 and Mouse anti-Nkx2.2: Coordination of progenitor specification and growth in mouse and chick spinal cord (Kicheva et al., 2014 Science DOI: 10.1126/science. 1254927)
Sheep anti-GFP : Id4 Downstream of Notch2 Maintains Neural Stem Cell Quiescence in the Adult Hippocampus (Zhang et al., 2019 Cell DOI: 10.1016/j.celrep.2019.07.014)
Rabbit anti-RFP: Cellular and molecular properties of neural progenitors in the developing mammalian hypothalamus (Zhou et al., 2020 Nature communications DOI: 10.1038/s41467-020-17890-2)
Rat anti-pH3: Cell intercalation driven by SMAD3 underlies secondary neural tube formation (Gonzalez-Gobartt et al., 2021 Developmental cell DOI: 10.1016/j.devcel.2021.03.023)
Goat anti-SOX2: Neural-specific Sox2 input and differential Gli-binding affinity provide context and positional information in Shh-directed neural patterning (Peterson et al., 2017, Genes & Dev DOI: 10.1101/gad.207142.112)
Rabbit anti-Brachyury: Defining the signalling determinants of a posterior ventral spinal cord identity in human neuromesodermal progenitor derivatives (Wind et al., 2021, Development DOI: 10.1242/dev.194415).

Animals and other research organisms

Policy information about studies involving animals; ARRIVE guidelines recommended for reporting animal research, and Sex and Gender in Research

Laboratory animals

Mus musculus embryos prior to midgestation were used. The following strains were bred to produce these embryos:
Tg(Sox2-cre/ERT2),
Gt(ROSA)26Sor<tm1(CAG-Brainbow2.1)Cle,
Gt(ROSA)26Sortm4(ACTB-tdTomato,-EGFP)Luo,
Igs2tm1(ACTB-EGFP,-tdTomato)Luo (MADM-11GT),
Igs2tm2(ACTB-tdTomato,-EGFP)Luo (MADM-11TG),
R26-ZO1-EGFP: Accession no. CDB0260K(http://www2.clst.riken.jp/arg/reporter_mice.html),
B6.Cg-Gt(ROSA)26Sortm9(CAG-tdTomato)Hze/J.

Wild animals

No wild animals were used.

Reporting on sex

The sex of the embryos used is unknown.

Field-collected samples	No field collected samples were used.
Ethics oversight	All animal procedures were performed in accordance with the relevant regulations and were approved under the license BMWFW-66.018/0006-WF/V/3b/2016 from the Austrian Bundesministerium für Wissenschaft, Forschung und Wirtschaft.

Note that full information on the approval of the study protocol must also be provided in the manuscript.
